# Dynamic calcium-mediated stress response and recovery signatures in the fungal pathogen, *Candida albicans*

**DOI:** 10.1128/mbio.01157-23

**Published:** 2023-09-26

**Authors:** C. V. Giuraniuc, C. Parkin, M. C. Almeida, M. Fricker, P. Shadmani, S. Nye, S. Wehmeier, S. Chawla, T. Bedekovic, L. Lehtovirta-Morley, D. M. Richards, N. A. Gow, A. C. Brand

**Affiliations:** 1 School of Medicine, Medical Sciences & Nutrition, University of Aberdeen, Aberdeen, United Kingdom; 2 MRC Centre for Medical Mycology at the University of Exeter, Exeter, United Kingdom; 3 School of Plant Sciences, University of Oxford, Oxford, United Kingdom; 4 Living Systems Institute, University of Exeter, Exeter, United Kingdom; 5 Department of Physics and Astronomy, University of Exeter, Exeter, United Kingdom; The University of British Columbia, Vancouver, British Columbia, Canada

**Keywords:** calcium signaling, calcineurin, stress response, *Candida albicans*, adaptation

## Abstract

Calcium (Ca^2+^) is an important second messenger for activating stress response signaling and cell adaptation in eukaryotic cells yet intracellular Ca^2+^-dynamics in fungi are poorly understood due to lack of effective real-time Ca^2+^ reporters. We engineered the GCaMP6f construct for use in the fungal pathogen, *Candida albicans*, and used live-cell imaging to observe both dynamic Ca^2+^ spiking and slower changes in non-spiking Ca^2+^-GCaMP signals elicited by stress or gene deletion. Short-term exposure to membrane, osmotic or oxidative stress generated immediate stress-specific responses and repeated exposure revealed differential recovery signatures. Osmotic stress caused yeast cell shrinkage and no adaptation response, where Ca^2+^-GCaMP spiking was inhibited by 1 M NaCl but not by 0.666 M CaCl_2._ Treatment with sodium dodecylsulfate (SDS) caused a spike-burst, raised the non-spiking Ca^2+^-GCaMP signals, and caused significant cell death, but surviving cells adapted over subsequent exposures. Treatment with 5 mM H_2_O_2_ abolished spiking and caused transient non-GCaMP-related autofluorescence, but cells adapted such that spiking returned and autofluorescence diminished on repeated exposure. Adaptation to H_2_O_2_ was dependent on Cap1, extracellular Ca^2+^, and calcineurin but not on its downstream target, Crz1. Ca^2+^-dynamics were not affected by H_2_O_2_ in the *hog1*Δ or *yvc1*Δ mutants, suggesting a pre-adapted, resistant state, possibly due to changes in membrane permeability. Live-cell imaging of Ca^2+^-GCaMP responses in individual cells has, therefore, revealed the dynamics of Ca^2+^-influx, signaling and homeostasis, and their role in the temporal stress response signatures of *C. albicans*.

**IMPORTANCE:**

Intracellular calcium signaling plays an important role in the resistance and adaptation to stresses encountered by fungal pathogens within the host. This study reports the optimization of the GCaMP fluorescent calcium reporter for live-cell imaging of dynamic calcium responses in single cells of the pathogen, *Candida albicans*, for the first time. Exposure to membrane, osmotic or oxidative stress generated both specific changes in single cell intracellular calcium spiking and longer calcium transients across the population. Repeated treatments showed that calcium dynamics become unaffected by some stresses but not others, consistent with known cell adaptation mechanisms. By expressing GCaMP in mutant strains and tracking the viability of individual cells over time, the relative contributions of key signaling pathways to calcium flux, stress adaptation, and cell death were demonstrated. This reporter, therefore, permits the study of calcium dynamics, homeostasis, and signaling in *C. albicans* at a previously unattainable level of detail.

## INTRODUCTION

*Candida albicans* is an opportunistic fungal pathogen that causes around 400,000 life-threatening bloodstream infections a year in patients undergoing immunosuppressive treatments, in addition to mucosal infections in millions of predisposed patients, particularly women of child-bearing age (1, 2). The regulation of mechanisms that contribute to pathogenesis by countering stresses caused by the host environment or by the action of antifungal drugs has been the focus of numerous studies. Calcium (Ca^2+^) is an essential trace element in all eukaryotic cells, where it acts as a co-factor in numerous enzyme functions but is also an important second messenger that activates cell stress responses. In *C. albicans*, this includes Ca^2+^ and membrane stress, which are key to its survival in serum and resistance to membrane-targeting antifungal drugs (3 – 5). Cytosolic [Ca^2+^]_cyt_ is maintained at ~80–120 nM (6) such that even small changes due to influx across the plasma-membrane or release from intracellular stores acts as an immediate stress signal. Ca^2+^-binding to calmodulin activates the calcineurin phosphatase that has many effectors but downstream activation of Crz1-dependent gene expression is the best-characterized to date (7).

Despite its importance in resilience to stress, the dynamics and regulation of Ca^2+^ flux in *C. albicans*, and indeed any fungus, are poorly understood due to the lack of effective tools with which to investigate changes both in real time and at the level of single cells. Studies have, therefore, been limited to those at the population level. For example, the ^45^Ca^2+^ radioisotope was used to demonstrate that the plasma-membrane Cch1-Mid1 channel is involved in Ca^2+^ uptake, and the photoprotein, aequorin, was used to show that cells swiftly take up Ca^2+^ in response to the detergent, SDS (8, 9). However, these methods yield no information on population heterogeneity, single-cell dynamics, or longer-term Ca^2+^ response signatures.

We have developed a new genetically encoded calcium indicator (GECI) based on the high signal-to-noise ratio, single-fluorophore indicator, GCaMP6 (10). The three variants of GCaMP6 (fast, medium, and slow) were originally developed for use in neurons, which maintain cytoplasmic [Ca^2+^] at 65–70 nM and have fast Ca^2+^ dynamics (10, 11). In *C. albicans*, cytoplasmic [Ca^2+^] is maintained at similar levels, but the dynamics of cytoplasmic Ca^2+^ flux are completely unknown. We selected the GCaMP6f variant and used it to investigate Ca^2+^ responses in *C. albicans* wild-type and signaling-pathway mutant cells when subjected to repeated exposure to stress compounds. Ca^2+^-GCaMP6 activity in resting cells appeared as cytoplasmic spikes of 5–6 s in duration whose frequency was dependent on extracellular [Ca^2+^] and an environmental pH of ≥7.0. In addition to spiking, some treatments generated raised non-spiking levels of cytoplasmic Ca^2+^-GCaMP signals, which were differentiated from changes in cell autofluorescence by comparing the signals from GCaMP-expressing cells with those observed in an empty-vector (non-GCaMP) control strain. Live-cell imaging of all three responses during treatment with cell stressors, including osmotic stress (1 M NaCl, 0.666 M CaCl_2_), membrane stress (0.05% SDS), and oxidative stress (5 mM H_2_O_2_), identified distinct stress-specific Ca^2+^-GCaMP6 signatures that suggest differential perturbation of plasma-membrane Ca^2+^-channel activity and intracellular Ca^2+^ homeostasis mechanisms. While osmotic stresses elicited the same response on each repeated treatment, Ca^2+^-dynamics slowly reverted to normal spiking and non-spiking behavior in the presence of SDS or H_2_O_2_, suggesting that cells were able to adapt. By expressing GCaMP in mutant strains, we showed that the Cap1 transcription factor, extracellular Ca^2+^, and the calcineurin phosphatase were required for adaptation to oxidative stress in *C. albicans*, but the calcineurin-dependent transcription factor, Crz1, was not. Nevertheless, deletion of Crz1 appeared to disorganize intracellular Ca^2+^ homeostasis. In contrast to wild-type cells, Ca^2+^-GCaMP spiking was not affected in the mutant lacking Hog1, a MAPK involved in both osmotic and oxidative stress pathways, suggesting that this mutant was pre-adapted to this stress. The GCaMP reporter, therefore, provides a novel method with which to investigate the regulation of Ca^2+^ homeostasis, stress-signaling, and adaptation over time, both at the population level and within individual cells.

## RESULTS

### The GCaMP6 response in *C. albicans* is [Ca^2+^]_ext_ and pH-dependent

**Fig 1:**
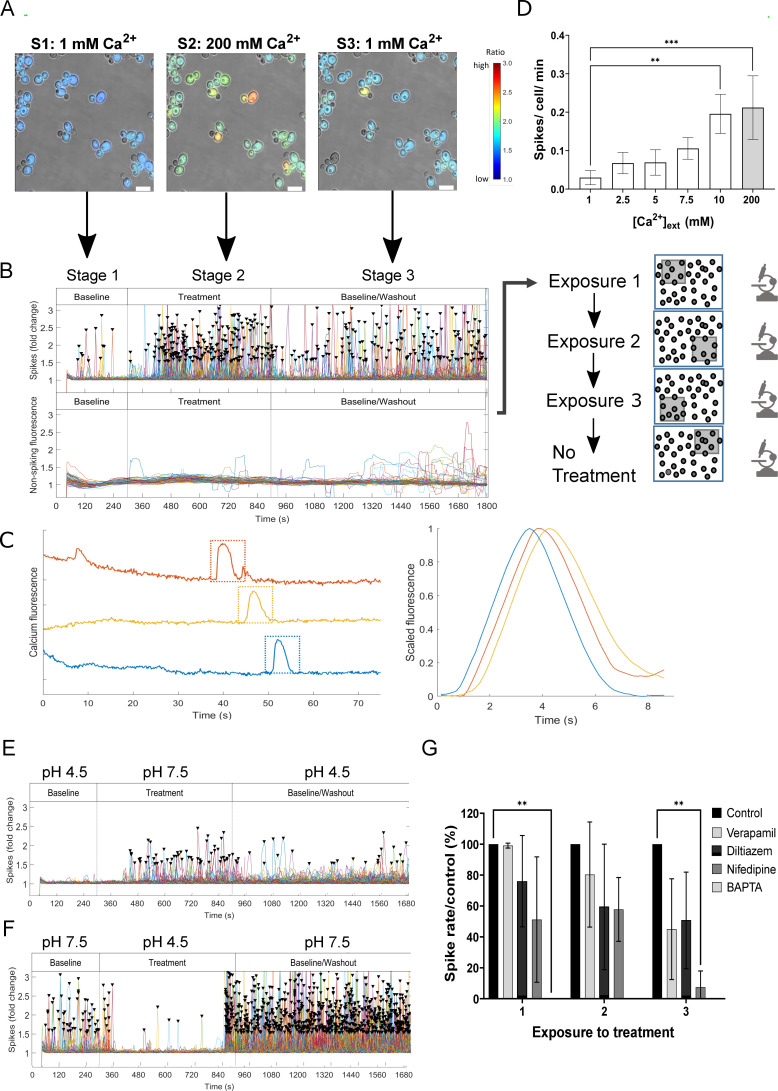
GCaMP6 spiking is dependent on [Ca^2+^]_ext_ and a neutral external pH. (**A**) Wild-type cells expressing GCaMP were imaged continuously during stage 1 (S1, 1 mM Ca^2+^), stage 2 (S2, 200 mM Ca^2+^), and stage 3 (S3, 1 mM Ca^2+^). This three-stage experiment constituted Exposure 1 (to 200 mM Ca^2+^). False colors reflect change in signal intensity (δ*F/F_b_* ratio). Bars = 10 µm. (**B**) Top panel—Ca^2+^-GCaMP spike distribution across the three stages of exposure 1. Each cell was assigned an arbitrary color. Bottom panel—non-spiking fluorescence signal over the same time-course. In later experiments, stages 1–3 were repeated three times such that cells received a total of three exposures to each stage 2 treatment, each at a different microscope position, followed by a no-treatment step to confirm cell growth. (**C**) Ca^2+^-GCaMP output imaged every 146 ms for 75 s. Left panel, output for three cells (colours are arbitrary). Right panel, shape of spikes (boxed in left panel) was analyzed with a bespoke MATLAB script that, after adjusting the contrast and correcting for photo-bleaching, calculated spike intensity as the mean of pixel intensity across each cell. (**D**) Spike rates generated by S2 treatments of increasing [Ca^2+^]_ext_. Error bars = mean ± SD, *n* = 3. Spike rates were compared to that observed in 1 mM Ca_2+_ using a one-way ANOVA with a Dunnett’s *post hoc t*-test. **P ≤ 0.01, ***P ≤ 0.001. (E) Ca^2+^-GCaMP spike output during the switch from pH 4.5 (stage 1) to pH 7.5 (stage 2) in the presence of 5 mM Ca^2+^. (**F**) The reverse experiment to that shown in panel **E**. (**G**) Comparison of effect of Ca^2+^-channel blockers on Ca^2+^-GCaMP spiking. From a stage 1 concentration of 1 mM Ca^2+^, at stage 2 cells were exposed to 5 mM Ca^2+^ and standardized concentrations (0.5 mM) of verapamil, diltiazem, or nifedipine, or 10 mM BAPTA. Ca^2+^ -GCaMP spikes/cell/min were expressed as a percentage of the spike rate of the no-blocker control. Only BAPTA (on the first exposure) and nifedipine (by the third exposure) produced a significant reduction in Ca^2+^-GCaMP-spiking. Error bars = mean ± SD. Data were analyzed using a two-way ANOVA comparing exposures and treatments, with a Dunnett’s *post-hoc t-*test against the no-treatment control. **P ≤ 0.01. Data include at least two biological replicates.

The GCaMP6f (fast) variant was synthesized to incorporate circularly permutated yEGFP3 along with the Ca^2+^-binding domain of calmodulin and the M13 peptide codon-optimized for *C. albicans* (10, 12) (Fig. S1). The construct was cloned into the CIpNAT and CIp10 plasmids (carrying nourseothricin resistance or the essential *URA3* gene as selectable markers, respectively) and transformed into gene deletion mutants and their relevant control strains (Tables S1 and S2). GCaMP6 activity was imaged in yeast cells in a CellASIC microfluidics plate perfused with trace-metal-free modified Soll’s medium (8) supplemented with CaCl_2_ (see below). Cells were acclimatized to these baseline conditions for 40 min by incubation at 30°C without imaging before commencement of a three-stage experiment. Stage 1 commenced with imaging in FITC and DIC every 5 s in baseline conditions for 5 min (Fig. 1A; Movie S1 [https://doi.org/10.5281/zenodo.8179089]). At stage 2, the perfusion channel was switched to MSM containing a compound of interest, to which cells were exposed for 10 min. This was followed by the stage 3 washout step and reversion to baseline conditions for 15 min. Cells were imaged continuously every 5 s at the same microscope position from the start of stage 1 to the end of stage 3 (a total of 30 min). These three stages constituted a single “exposure” to a treatment. In adaptation experiments, the three-stage process was repeated at microscope positions 2 and 3, which allowed quantification of the effect on a cell population of three exposures to the same treatment while minimizing photo-stress (Fig. 1B). Continued cell growth was confirmed by imaging at the fourth position in DIC only, without treatment. The imaging output of each cell per field of view over time was analyzed by custom software (see Materials and Methods). The intensity of Ca^2+^ transients was depicted in each cell by converting the change in fluorescence ratio value (*F/F_b_*) to a rainbow color “heat map” scale running from blue to red. In the output plots, the signal from each cell was assigned an arbitrary color to aid individual cell-tracking over time. This analysis revealed two modes of Ca^2+^-GCaMP response: dynamic fluorescent cytoplasmic spiking (plotted as spikes/cell/min, a metric that overcame variation in the number of cells per field of view) and longer, non-spiking Ca^2+^-GCaMP signals, denoting slower changes in GCaMP-derived fluorescence above stage 1 values. The non-spiking Ca^2+^-GCaMP signal was differentiated from cell autofluorescence by comparing the fluorescence outputs between strains transformed with either the GCaMP construct or the empty-vector as a no-GCaMP control. Fast-acquisition imaging at 146 ms showed that spike duration was 5–6 s (Fig. 1C). However, imaging at this frame rate caused photo-stress and significant cell death within 15 min. A frame rate of 5 s was therefore used as it permitted informative imaging at each microscope position for 30 min without inducing significant photo stress during the period of interest (Fig. S2) or affecting cell viability (Fig. S3).

Investigation of the relationship between extracellular calcium, [Ca^2+^]_ext_, and Ca^2+^-GCaMP responses showed that spiking frequency positively correlated with [Ca^2+^]_ext_. Within the range of 1–10 mM, values increased from 0.03 to 0.2 spikes/cell/min (Fig. 1D) but did not significantly increase further on exposure to 200 mM Ca^2+^. Ca^2+^-GCaMP spiking in the presence of 5 mM [Ca^2+^]_ext_ was completely abolished by the addition of 10 mM BAPTA, a membrane-impermeable, Ca^2+^-specific chelator, indicating that spiking resulted from Ca^2+-^influx through the plasma-membrane (Fig. 1G). Changes in [Ca^2+^]_ext_ from 1 to 200 mM did not affect non-spiking Ca^2+^-GCaMP levels, suggesting that cellular Ca^2+^-homeostasis mechanisms adequately maintained cytoplasmic [Ca^2+^] in these conditions (Fig. 1B).

We next investigated the optimum extracellular pH at which to visualize output from the GCaMP construct. The aequorin Ca^2+^ reporter in *C. albicans* requires a pH of ~7 or above (9). To test whether this was also true for GCaMP, the pH in stage 1 was set at either 4.5 or 7.5 and the pH value was reversed during stage 2. During stage 1 at pH 4.5, spiking was negligible but commenced at a rate of 0.026 ± 0.019 spikes/cell/min on the switch to pH 7.5 in stage 2 (Fig. 1E). In the reverse experiment when the stage 1 baseline was set at pH 7.5, spike rate became negligible on the switch to pH 4.5 at stage 2 (Fig. 1F). Therefore, as seen for aequorin, optimum activity of the GCaMP reporter was observed when the external pH was maintained at, or slightly above, neutral. Taken together with the finding in *Saccharomyces cerevisiae* that during exposure to external pH values between pH 3 and 8, the intracellular pH was maintained at pH 7–7.5 (13, 14) the requirement for neutral pH for imaging Ca^2+^-influx appears to be linked to the biology of the cell and not to a property of the reporter.

These experiments defined optimal conditions in which to characterize the immediate Ca^2+^-GCaMP response in *C. albicans* yeast cells to commonly applied *in vitro* stress treatments. All further experiments were, therefore, conducted at pH 7.5 with a stage 1 [Ca^2+^]_ext_ of 5 mM and an imaging frame rate of 5 s as these parameters adequately captured changes in spiking frequency between stages 1 (baseline), 2 (treatment), and 3 (washout) without causing significant photo-stress.

We next examined the ability of three clinic-based inhibitors that have commonly been used with the intention of blocking Ca^2+^influx through the L-type Ca^2+^-channel homolog in *C. albicans,* Cch1. For comparative purposes, equimolar concentrations (0.5 mM) of the two non-dihydropyridines, diltiazem (a benzothiazepine) and verapamil (a phenylalkylamine), or the dihydropyridine, nifedipine, were added at stage 2 where the [Ca^2+^]_ext_ was simultaneously increased from 1 to 5 mM. At the concentrations used, none of the channel-blockers abolished Ca^2+^-GCaMP spiking during the first 10 min exposure but instead progressively inhibited spiking upon each subsequent treatment (Fig. 1G). Verapamil had the slowest kinetics and reduced spiking to ~50% of the control by treatment 3, ultimately achieving the same level of inhibition as diltiazem. The finding that verapamil does not completely inhibit Ca^2+^-uptake is consistent with previous findings in *S. cerevisiae* (15) and in *C. albicans* (8). Nifedipine was the only clinical channel-blocker to significantly reduce Ca^2+^-spiking (by 92.5% compared to the control strain, *P* ≤ 0.01), a level that was reached on the third exposure. However, at 0.5 mM, none of the inhibitors was as effective at blocking Ca^2+^-GCaMP spiking as the use of 10 mM BAPTA (i.e., a 2:1 ratio with [Ca^2+^]_ext_), which completely abolished spiking during the first exposure (*P* ≤ 0.01).

### Equimolar osmotic shock caused cell shrinkage, but Ca^2+^-GCaMP responses were ion-dependent

**Fig 2:**
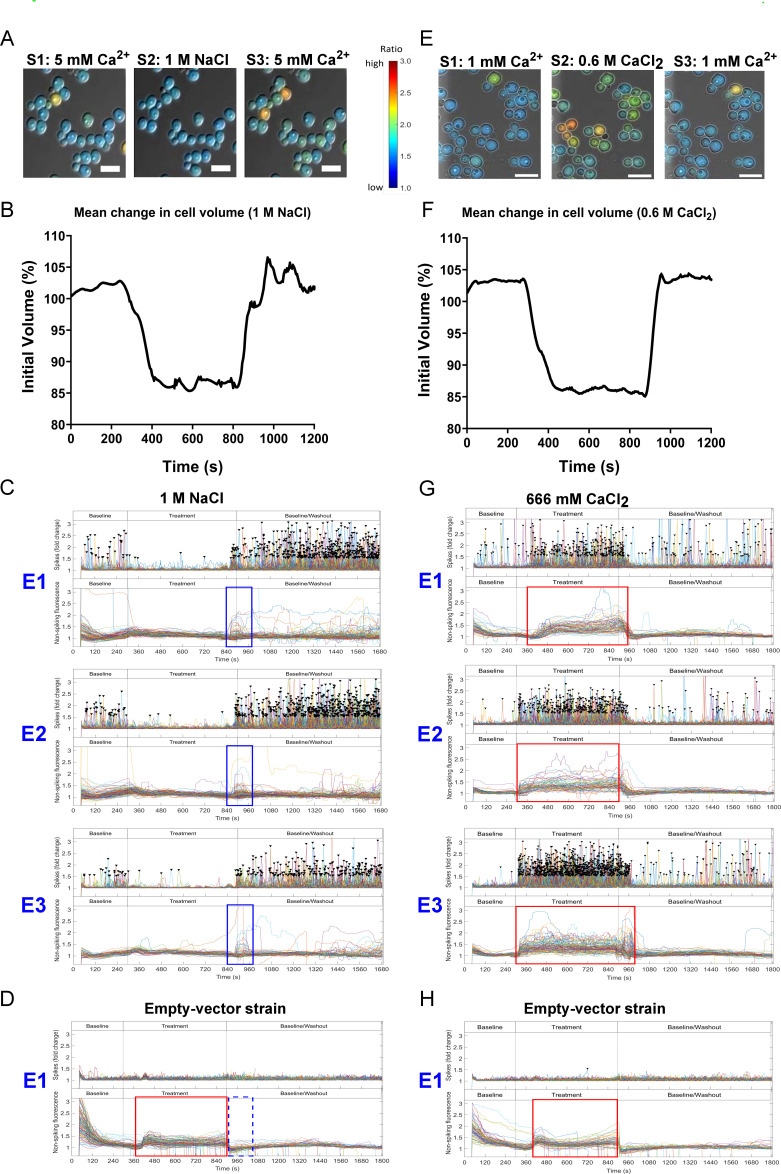
Equi-molar osmotic shocks caused cell shrinkage and differential ion-dependent Ca^2+^-GCaMP responses. (**A**) Ca^2+^-GCaMP imaging in response to 1 NaCl. (**B**) Effect of 1 M NaCl on cell volume. The volume of all cells per field of view at each time point was normalized to the initial volume (*n* = 3). (C–G) Blue box highlights presence (solid line) or absence (broken line) of a GCaMP-dependent signal. Red box highlights presence (solid line) or absence (broken line) of autofluorescence (GCaMP-independent). (**C**) Exposure to 1 M NaCl shuts down GCaMP Ca^2+^ spiking. E1–E3 = exposures 1–3. Top panels: Ca^2+^-GCaMP spike output. Bottom panels: non-spiking fluorescence. A rise in non-spiking GCaMP-dependent fluorescence is highlighted (blue box). (**D**) Ca^2+^-GCaMP-dependent signals were absent in the empty-vector (A569) strain, but there was a slight rise in autofluorescence during exposure to 1 M NaCl (red box). (**E**) Ca^2+^-GCaMP imaging in response to 0.666 M CaCl_2._ (**F**) Effect of 0.666 M CaCl_2_ on cell volume. (**G**) Exposure to 0.666 M CaCl_2_. Ca^2+^-GCaMP increased spike rate (top panels) and caused a slight rise in non-spiking autofluorescence (red box). (**H**) No Ca^2+^-GCaMP spiking was seen in the empty-vector strain but there was a slight rise in autofluorescence (red box) in the presence of 0.666 M CaCl_2_. Size bars = 10 µm.

Ca^2+^-signaling is involved in a number of stress responses in *C. albicans*. We therefore imaged Ca^2+^-GCaMP dynamics during the application of a stress compound at stage 2 for 10 min, followed by washout (stage 3). The exposure was repeated at two further microscope positions to look for stress adaptation. To test osmotic stress, cells were exposed to equivalent ionic concentrations (2 osmol/L) of either 1 M NaCl or 0.666 M CaCl_2_. During the first exposure to 1 M NaCl, cell volume shrank by ~22%, as observed previously (16), and Ca^2+^-GCaMP6 spiking almost ceased (Fig. 2A, B, and C; Movie S2 [https://doi.org/10.5281/zenodo.8179089]). On commencement of stage 3 (washout), cells regained their initial volume and spiking recommenced with a short period (~2 min) of intense spiking before resumption of a steady rate. This was accompanied by a transient rise in non-spiking Ca^2+^-GCaMP signals, which was not seen in the strain transformed with the empty vector (Fig. 2C and D, blue boxes). Similar results were seen during the two further exposures to 1 M NaCl, suggesting that there was an intense influx of Ca^2+^ on removal of high [NaCl]. Imaging of the empty-vector strain showed that 1 M NaCl produced a slight increase in autofluorescence during the treatment phase, but this disappeared immediately on commencement of the stage 3 washout. Despite the loss of cell volume, viability was unaffected by this short period of osmotic shock as Ca^2+^-spiking recommenced and cells were PI-negative after three treatments (Fig. S3A).

When the experiment was repeated using 0.666 M CaCl_2_ as an alternative and equimolar salt stress, cell volume decreased by ~17%, indicating that shrinkage is a general response to osmotic stress (Fig. 2E and F; Movie S3 [https://doi.org/10.5281/zenodo.8179089]). However, in contrast to the spiking shut-down seen in response to NaCl, the spike rate increased 6.3-fold over baseline in the presence of high [CaCl_2_]_ext_, indicating that Ca^2+^ channels retained function during cell shrinkage. Loss of Ca^2+^-GCaMP6 spiking in the presence of 1 M NaCl was therefore likely due to the high external Na^+^:Ca^2+^ ratio in which Ca^2+^ entry was blocked and is consistent with the short, population-wide rise in the non-spiking Ca^2+^-GCaMP signal at commencement of NaCl washout. Exposure to 0.666 mM CaCl_2_ elicited the same level of autofluorescence in the empty-vector strain as observed for 1 M NaCl (Fig. 2D, G, and H, red boxes), suggesting that autofluorescence and cell shrinkage are general cell responses to osmotic stress and are not ion-specific.

### Exposure to SDS elicits a three-phase Ca^2+^ signature accompanied by major cell death and adaptation in the surviving population

**Fig 3:**
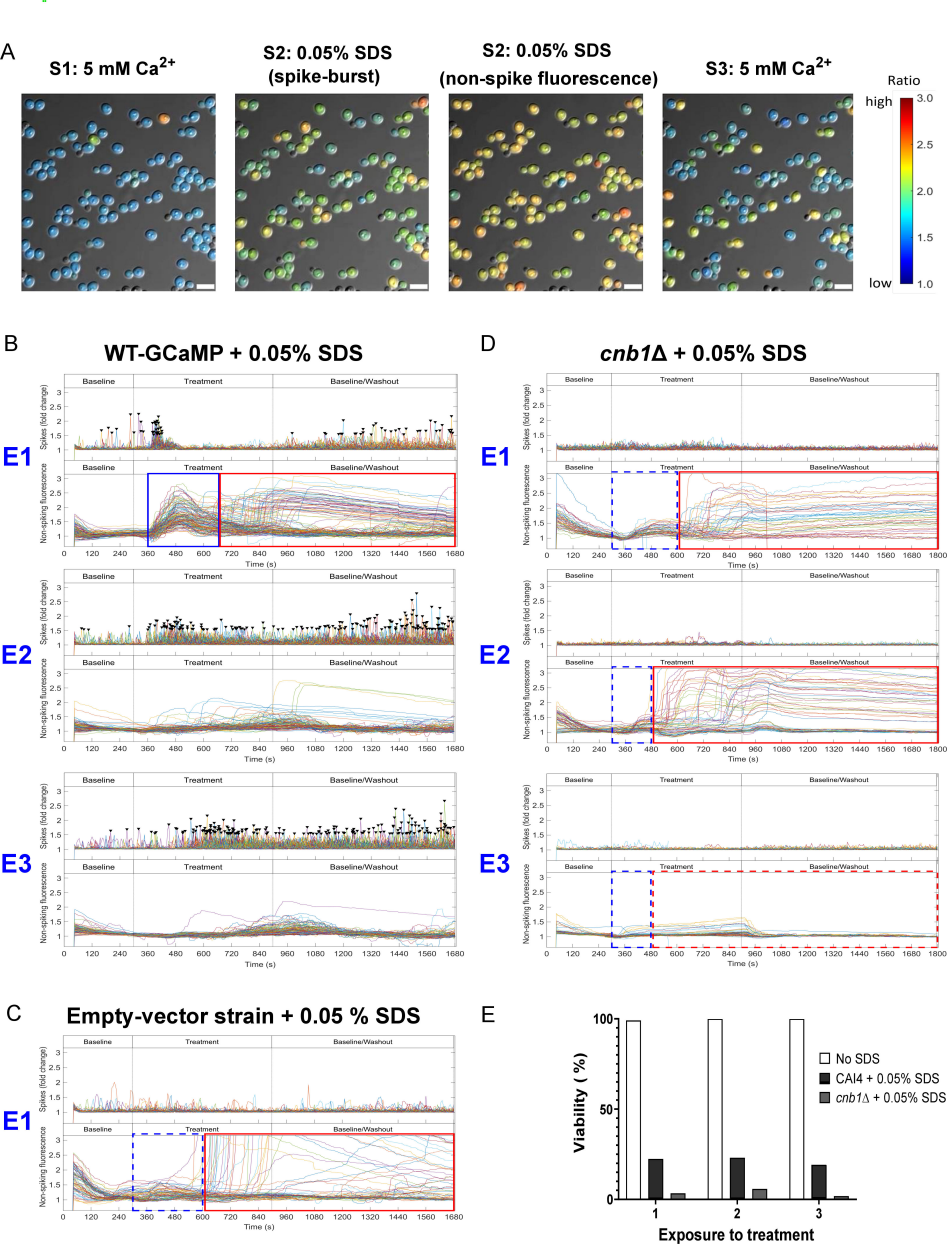
Exposure to SDS causes a three-phase response and significant cell death but surviving cells undergo calcineurin-dependent adaptation upon repeat exposure. (**A**) Ca^2+^-GCaMP imaging revealed a three-phase response to 0.05% SDS. (**B**) Imaging output plots of WT-GCaMP across three exposures to SDS. E1–E3 = exposures 1–3. After a spike-burst, a synchronous rise in non-spiking GCaMP signal (blue box) was followed by non-synchronous autofluorescence (red box). (**C**) Fluorescence output of the empty-vector strain showing absence of the non-spiking GCaMP signal (blue box, broken line) and asynchronous autofluorescence (red box). (**D**) Ca^2+^-GCaMP output plots of the *cnb1*Δ-GCaMP strain. (**E**) Effect on cell viability of exposure to SDS in the control strain and *cnb1*Δ-GCaMP. Cells were stained with 1 µg/mL propidium iodide (PI) at the end of E3 and % viability expressed as the number of PI positive cells as a % of total cells at each position compared to non-exposed CAI4–GCaMP. Scale bars = 10 µm.

The anionic surfactant, sodium dodecylsulfate (SDS), de-stabilizes plasma-membrane integrity, which activates the Ca^2+^-calcineurin signaling pathway (4). In our system, the first exposure to SDS (0.05%) caused an intense, but short-lived, 40–60 s Ca^2+^-GCaMP spike-burst (Fig. 3A; Movie S4 [https://doi.org/10.5281/zenodo.8179089]). This was immediately followed by a synchronous rise in the non-spiking Ca^2+^-GCaMP signal, which was not seen in the empty-vector strain (Fig. 3B and C, blue boxes). The rise could be due to unregulated Ca^2+^-influx across the compromised plasma-membrane. There was no further spiking after the initial burst and the non-spiking Ca^2+^-GCaMP signal gradually declined during the 10 min treatment, suggesting the activity of cytosolic Ca^2+^ homeostasis regulators that remove Ca^2+^ from the cytoplasm. During the subsequent two exposures to SDS, the initial spike-burst reduced in intensity and lengthened in duration, with no reappearance of the synchronous non-spiking Ca^2+^-GCaMP signal. After the initial first shock, the cell response appeared to be one of gradual recovery on subsequent treatments with SDS.

During the first exposure to SDS, just as the non-spiking Ca^2+^-GCaMP signal was declining, a large number of cells underwent an asynchronous and significant long-term rise in autofluorescence, which was identified as such because it was also present in the empty-vector strain on treatment with SDS (Fig. 3B and C, red boxes). Propidium iodide (PI) was flowed into the system after exposure 3 and the fluorescent output of individual cells at each microscope position was compared to their end-point viability status. This revealed that only 20% of the cells that exhibited spiking activity prior to the first exposure to SDS survived all three exposures. The other 80% of the population were the high-autofluorescence cells and were PI positive at the end of the experiment (Fig. 3E). Cell death was dependent on the presence of extracellular [Ca^2+^], as the addition of BAPTA during SDS treatment rescued cell viability (Fig. S3B). However, the number of spiking cells that underwent cell death decreased at each exposure and 98% of cells that were still spiking after exposure 2 survived the final treatment. The gradual reduction in the severity of the spike-burst, the rise in non-spiking Ca^2+^-GCaMP signals and the asynchronous generation of autofluorescence across the three exposures to SDS suggest that, while the majority of the cell population did not survive the first exposure, those that did were either pre-resistant or underwent an adaptation process that conferred resistance. However, while spiking was indicative of cell viability, spiking activity was not, in itself, essential for cell survival in SDS as 7% of non-spiking cells were nevertheless viable after exposure 3.

The phosphatase, calcineurin, is required for plasma-membrane integrity, so we next compared these responses with those of the *cnb1*Δ null mutant. The *cnb1*Δ-GCaMP strain produced few and weak spikes (see below) and no rise in non-spiking Ca^2+^-GCaMP signals during the first exposure to SDS, unlike the WT-GCaMP strain (Fig. 3D, blue boxes), indicating that calcineurin contributes to the generation of Ca^2+^-GCaMP signals. No spikes were seen during stage 1 but more cells exhibited asynchronous autofluorescence on Exposures 1 and 2 to SDS (Fig. 3D, red boxes) compared to the empty-vector strain (Fig. 3C). All the *cnb1*Δ-GCaMP cells were PI positive after three exposures to SDS so the lack of spiking, coupled with the failure of cells to autofluorescence during exposure 3, strongly suggests that the entire population had lost viability during exposures 1 and 2 (Fig. 3E), confirming that calcineurin is required for resistance to membrane stress.

### *C. albicans* adaptation to repeated treatment with 5 mM H_2_O_2_

**Fig 4:**
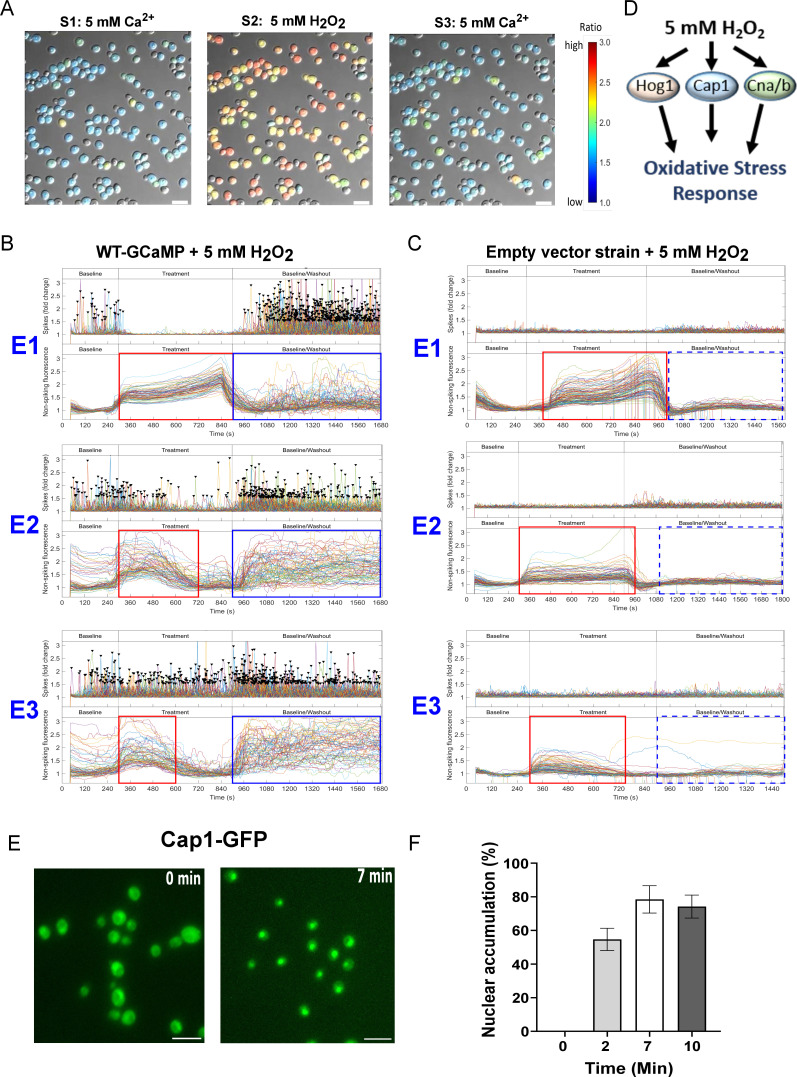
The inhibition of Ca^2+^-GCaMP spiking on exposure to 5 mM H_2_O_2_ is ameliorated during repeated treatments. (**A**) Imaging of WT-GCaMP cells during exposure to 5 mM H_2_O_2_. (**B, C**) The presence (solid lines) or absence (broken lines) of GCaMP-dependent signals (blue box) or autofluorescence (red box) is highlighted. (**B**) Imaging output plots of WT-GCaMP across three exposures to H_2_O_2._ Top panels = Ca^2+^-GCaMP spiking. Bottom panels = nonspiking-spiking fluorescence. E1–E3 = exposures 1–3. (**C**) Fluorescence outputs of empty-vector strain when treated with 5 mM H_2_O_2_. (**D**) Putative signaling pathways involved in the oxidative stress response in *C. albicans*. (**E**) Cap1-GFP localization at *t* = 0 and *t* = 7 min on cell exposure to 5 mM H_2_O_2_. Bars = 10 µM. (**F**) Time course of Cap1-GFP translocation into the nucleus, imaged every 60 s after exposure to 5 mM H_2_O_2_. Bars = mean ± SD (*n* = 2: 220 cells examined).

*C. albicans* is exposed to reactive oxygen species (ROS) in the phagolysosome of immune cells (17, 18), so we examined the Ca^2+^-GCaMP response to oxidative stress using 5 mM H_2_O_2_. In our system, the first exposure to H_2_O_2_ inhibited Ca^2+^-GCaMP spiking within ~1 min and was accompanied by a population-wide rise in autofluorescence, which was also observed in the empty-vector strain, for the duration of the treatment (Fig. 4A, B, and C, red boxes; Movies S5 and S6 [https://doi.org/10.5281/zenodo.8179089]). On removal of H_2_O_2_, spiking re-commenced and the autofluorescence declined. During the 15-min washout phase (stage 3), a proportion of cells underwent asynchronous short-term increases in the non-spiking signal that were not seen in the empty-vector control, suggesting that these signals were Ca^2+^-GCaMP-dependent and that the cells had raised [Ca^2+^]_cyt_ ( Fig. 4B and C, blue boxes). However, unlike the high cell death linked to asynchronous autofluorescence seen during SDS treatment, the population displaying asynchronous, non-spiking Ca^2+^-GCaMP-signals remained viable throughout the three exposures to H_2_O_2_ (Fig. S3C). During the second exposure to H_2_O_2_, inhibition of Ca^2+^-GCaMP spiking was only partial and was not seen on the third exposure, where Ca^2+^-GCaMP spiking continued throughout. Furthermore, the autofluorescence rises seen in both GCaMP-expressing cells and the empty-vector strain faded with successive treatments. This suggests that Ca^2+^-spiking and the intracellular chemistry responsible for autofluorescence were modified by an adaptation response driven by gene expression during the 90-min time course of the experiment, such that H_2_O_2_ no longer elicited a shock response. In support of this, we confirmed that the Cap1 transcription factor, a key driver of the Oxidative Stress Response (OSR) in *C. albicans* (19), translocated from the cytoplasm to the nucleus in 55% of cells within 2 min and 78% of cells within 7 min of exposure to H_2_O_2_ in our experimental system (Fig. 4E and F), consistent with previous findings (20, 21). This supports the idea that amelioration of the initial shock response was due to cell adaptation that was driven by gene expression.

### Adaptation to H_2_O_2_ requires the Cap1 transcription factor, but *hog1*Δ cells appear pre-adapted

**Fig 5:**
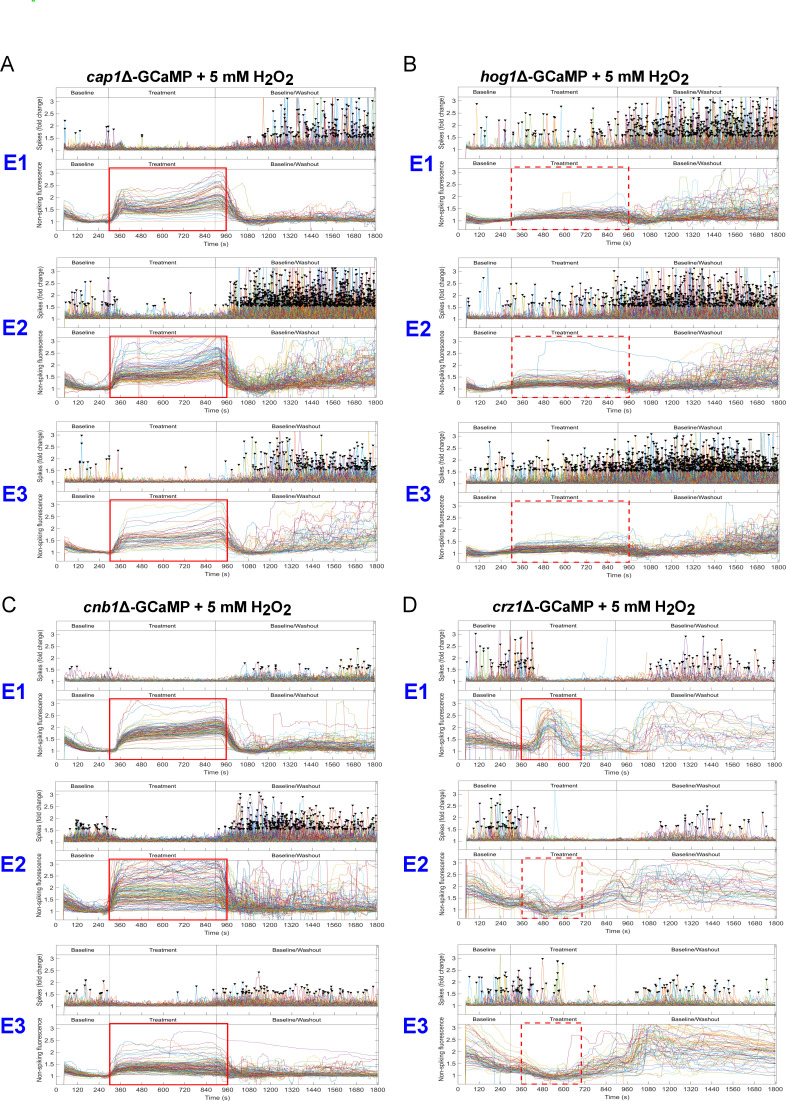
Restoration of Ca^2+^-GCaMP spiking in 5 mM H_2_O_2_ requires Cap1 and calcineurin and is only partially dependent on the Crz1 transcription factor. Spiking was not inhibited in *hog1*Δ. Ca^2+^ GCaMP spike (top panels) distributions and non-spiking fluorescence (bottom panels) of cells exposed to 5 mM H_2_O_2_ across the three-stage time course during exposures 1–3 (**E1–E3**). The presence (solid line) or absence (broken line) of autofluorescence is highlighted in red. (A) *cap1∆*-GCaMP, (B) *hog1∆*-GCaMP, (C) *cnb1∆*-GCaMP, and (D) *crz1∆*-GCaMP.

The OSR in *C. albicans* is mediated by two signaling pathways—the Cap1 transcription factor and the Hog1 kinase (Fig. 4D) (20, 22 – 24). We examined whether these pathways were required for the restoration of Ca^2+^ influx during exposure to H_2_O_2_ by expressing GCaMP6 in the respective gene deletion mutants. The first exposure of *cap1*Δ-GCaMP to H_2_O_2_ yielded a shutdown in Ca^2+^-GCaMP spiking and the generation of autofluorescence similar to the response of the wild-type-GCaMP strain (Fig. 5A, red boxes; Movie S7 [https://doi.org/10.5281/zenodo.8179089]). However, the subsequent amelioration of these responses observed in the wild-type-GCaMP strain during treatments 2 and 3 was absent in the *cap1*Δ mutant, indicating that Cap1 was required for the adaptive response that permitted continued Ca^2+^-GCaMP activity during oxidative stress.

The MAPK, Hog1 (high-osmolarity glycerol 1), is phosphorylated and accumulates in the nucleus during oxidative stress, where it confers resistance to high levels of ROS (22, 25), although its effectors do not appear to include transcription factors (26). Remarkably, in our system, unlike the shutoff of Ca^2+^-GCaMP activity seen in control cells during exposure 1, the *hog1*Δ-GCaMP strain was minimally affected by 5 mM H_2_O_2_ as spiking continued and autofluorescence was minimal (Fig. 5B, red boxes; Movie S8 [https://doi.org/10.5281/zenodo.8179089]). Treatments 2 and 3 elicited increasingly milder responses. The slight shifts seen on washout (an increase in spike rate and decrease in autofluorescence) indicate that cells were able to sense the treatment, but the overall response profile was very similar to that seen during the “adapted” response in the wild-type-GCaMP strain. As the response of the *hog1*Δ-GCaMP mutant during each exposure to H_2_O_2_ was the same as the adapted response signature seen in wild-type cells (i.e., Ca^2+^ spiking continued and there was no significant rise in autofluorescence), it appears that the OSR was already pre-activated in the mutant. It also suggests that Hog1 acts as a repressor of the OSR in wild-type cells.

### Adaptation to oxidative stress requires calcineurin but is only partially dependent on the transcription factor, Crz1

A third pathway proposed to be involved in the response to oxidative stress is regulated by the phosphatase, calcineurin. Calcineurin function has been best characterized in terms of its role in combatting membrane and Ca^2+^-stress, and its activation of gene expression via the Crz1 transcription factor (3, 4, 7). We examined the importance of calcineurin to the OSR using the *cnb1*Δ-GCaMP strain, which lacks the regulatory subunit. During Exposure 1, the response signature to H_2_O_2_ was very similar to that seen in *cap1*Δ, where spiking ceased and autofluorescence remained elevated throughout the treatment period (Fig. 5C, red boxes; Movie S9 [https://doi.org/10.5281/zenodo.8179089]). As in the *cap1*Δ mutant, there was no major change in the Ca^2+^-GCaMP6 or autofluorescence signature at treatments 2 and 3, suggesting that cells were unable to adapt to H_2_O_2_. Calcineurin, therefore, plays a role of equal importance to that of Cap1 in restoring Ca^2+^-flux and reducing autofluorescence in the presence of oxidative stress.

To further probe the contribution of calcineurin-associated effectors to the OSR, GCaMP was expressed in the *crz1*Δ null mutant. In *crz1*Δ, the Ca^2+^-GCaMP spiking signature on treatment 1 was somewhat erratic but essentially the same as that seen in the wild-type-GCaMP strain, with a cessation in spiking and a rise in autofluorescence (Fig. 5D, red boxes; Movie S10 [https://doi.org/10.5281/zenodo.8179089]). However, unlike the *cnb1*Δ mutant, Ca^2+^ spiking resumed and autofluorescence reduced during subsequent treatments. In addition, as in the wild-type-GCaMP strain, there was a rise in asynchronous non-spiking Ca^2+^-GCaMP signals during the washout phase. Therefore, even though non-spiking Ca^2+^-GCaMP signals became increasingly disorganized, it appeared that the *crz1*Δ mutant was able to undergo adaptation to oxidative stress. Crz1 is responsible for transcription of 69 calcineurin-dependent genes, 5 of which (including Golgi Pmr1 and vacuolar Pmc1) are required for Ca^2+^ transport and homeostasis (7, 27), so it is possible that the erratic Ca^2+^-GCaMP signature seen on Crz1 deletion was due to the cells’ inability to regulate homeostasis during onset of the stress response. The finding that calcineurin, but not Crz1, is required for adaptation points to the importance of alternative calcineurin effectors for delivering a normal response to oxidative stress.

### The plasma-membrane Ca^2+^-channel, Cch1, and extracellular Ca^2+^, but not the vacuolar TRP-channel, Yvc1, is required for adaption to oxidative stress

**Fig 6:**
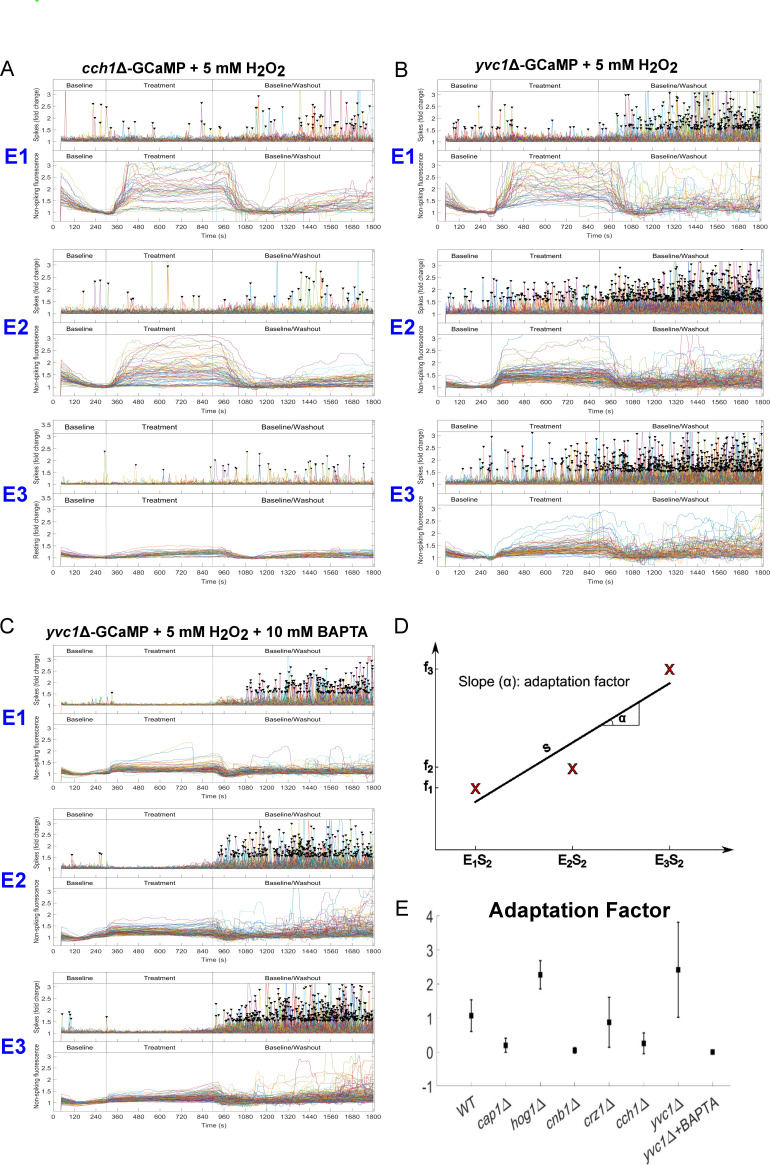
Cch1 and extracellular Ca^2+^, but not Yvc1, are required for adaptation to H_2_O_2_ treatment. Ca^2+^ GCaMP spike (top panels) distributions and non-spiking fluorescence (bottom panels) of cells exposed to 5 mM H_2_O_2_, across the three stage time course during exposures 1–3 (**E1–E3**). (**A**) *cch1∆*-GCaMP, (**B**) *yvc1∆*-GCaMP, and (C) *yvc1∆*-GCaMP + 10 mM BAPTA. (**D**) Definition of the adaptation factor (AF) as the gradient of the slope plotted onto the spike frequency per cell at each of the three exposures to H_2_O_2_. (**E**) Adaptation factors of wild-type and mutant strains on treatment with 5 mM H_2_O_2_. Student’s *t*-tests (with Bonferroni correction for multiple comparisons) show that the wild type, *hog1*∆, *crz1*∆, and *yvc1*∆ adapt to H_2_O_2_ (AF significantly different to 0), whereas *cap1∆*, *cnb1∆*, *cch1∆,* and *yvc1∆* + BAPTA do not (AF not significantly different to 0).

Influx of Ca^2+^ into the cytoplasm requires entry through the plasma-membrane from the extracellular environment or release from intracellular stores. Currently, the only known channels that mediate cytoplasmic Ca^2+^ inflow are plasma-membrane-localized Cch1, which is most closely related to non-voltage-gated sodium leak channels (NALCN), and Yvc1, a TRP channel that releases Ca^2+^ from the vacuole (28, 29). We first undertook a comparison of the Ca^2+^-GCaMP resting spike rates in *cch1*Δ, *yvc1*Δ, and *cnb1*Δ cells during the first stage 1 period prior to any treatment, which revealed significant differences between the mutants and wild-type cells (Fig. S4). The Ca^2+^ spike rate was lower in resting *cch1*Δ-GCaMP cells compared to wild-type cells. However, Ca^2+^-spiking was not abolished, indicating that Ca^2+^ was able to enter the cytoplasm in this mutant. Further investigation showed that spiking increased in *cch1*Δ-GCaMP when [Ca^2+^]_ext_ was raised to 200 mM Ca^2+^ (Fig. S5) although the fold increase was under half that seen in wild-type cells (3.6- vs 8.4-fold, respectively), but this, nevertheless, suggested that spiking was driven by Ca^2+^ influx via an alternative plasma-membrane Ca^2+^-channel as opposed to vacuolar efflux via Yvc1. Spiking was also significantly reduced in the *yvc1*Δ-GCaMP strain and almost abolished in *cnb1*Δ-GCaMP, indicating that both vacuolar Yvc1 and calcineurin positively regulate Ca^2+^-influx through plasma-membrane channels, with calcineurin regulating not only Cch1 but all Ca^2+^ influx channels. However, initial spike rate was not an indicator of the ability of cells to adapt to H_2_O_2_ as, although their resting spike rates were both approximately half that of wild-type cells, the *yvc1*Δ mutant exhibited adaptation but *cch1*Δ did not (Fig. 6A; Movie S11 [https://doi.org/10.5281/zenodo.8179089]). However, in *cch1*Δ, the high level of synchronous autofluorescence seen on exposure 1 declined to negligible by exposure 3, indicating that Cch1 is not involved in this aspect of adaptation to H_2_O_2_. Interestingly, the asynchronous rises in non-spiking Ca^2+^-GCaMP signals that were seen during stage 3 in the wild-type-GCaMP strain were muted throughout in *cch1*Δ-GCaMP cells, suggesting that they are dependent on Cch1 and result from dysregulated Ca^2+^ entry. In the *yvc1*Δ-GCaMP strain, the response and adaptation to repeated H_2_O_2_ exposure were very similar to that seen in the wild-type-GCaMP strain, although, like the *hog1*Δ mutant, Ca^2+^-spiking was not completely shut down in *yvc1*Δ (Fig. 6B; Movie S12 [https://doi.org/10.5281/zenodo.8179089]). However, when extracellular Ca^2+^ was chelated by BAPTA, it was completely abolished and the perturbation in autofluorescence in *yvc1*Δ-GCaMP was also minimal (Fig. 6C). Therefore, Ca^2+^ release from the vacuole via Yvc1 does not contribute to Ca^2+^-GCaMP spiking but may alter the intracellular chemistry that generates synchronous autofluorescence during oxidative stress. Taken together, these results suggest that there is a low-conductance plasma-membrane channel in addition to high-conductance Cch1 in *C. albicans*, which is consistent with the proposed existence of a low-affinity calcium uptake system and high-affinity calcium uptake system (6, 30 – 32). In the absence of Cch1, this low-conductance channel was not rescued from its partial inhibition on repeated exposure to H_2_O_2_. Neither Cch1 or Yvc1 play a role in the rise or adaptive recovery of synchronous autofluorescence on repeated treatments, which occurred in wild-type, *cch1*Δ and *yvc1*Δ mutants, but not the *cap1*Δ and *cnb1*Δ strains.

### Use of an “adaptation factor” to characterize the restoration of Ca^2+^-GCaMP spiking on repeated exposure to H_2_O_2_ in signaling pathway mutants

In order to compare adaptation responses in the wild-type strain and signaling pathway mutants to repeated H_2_O_2_ exposure, the mean Ca^2+^-GCaMP spike rate during each stage 2 treatment period was plotted across the three exposures to H_2_O_2_ for each strain (Fig. 6D and E; Fig. S6), and the adaptation factor determined by linear regression. The *cap1*Δ and *cnb1*Δ mutants showed no restoration of Ca^2+^-GCaMP spiking after the shutoff seen during the first exposure to H_2_O_2,_ unlike the wild-type strain and the *crz1*Δ and *yvc1*Δ mutants (Fig. 6E). Ca^2+^-spiking was not completely abolished in *cch1*Δ-GCaMP but did not recover over successive H_2_O_2_ treatments, indicating that Cch1 is required for adaptation. The *hog1*Δ mutant adapted but from a different starting position compared to the control strain, as spiking in this mutant was not inhibited on the first exposure H_2_O_2_. However, strains that underwent adaptation to H_2_O_2_ were differentially affected in other ways, for example, autofluorescence commenced at a high level in *yvc1*Δ but reduced over time, while in the *hog1*Δ mutant, the autofluorescence level was minimal throughout. Therefore, Ca^2+^-GCaMP spiking behavior is a valuable indicator of cell viability and active homeostasis mechanisms, while non-spiking GCaMP and autofluorescence signals provide additional and distinct insights into the physiological aspects of cell responses.

### Exposure to the antifungal drugs caspofungin and fluconazole does not elicit an immediate Ca^2+^ stress response

Cell mechanisms that confer resistance to antifungal drugs are of great interest in medical mycology. We therefore used our system to test whether exposure to fluconazole (8 µg/mL), which targets ergosterol biosynthesis, or caspofungin (0.032 µg/mL), which inhibits cell-wall biosynthesis, elicit changes in Ca^2+^-GCaMP6 activity. Wild-type-GCaMP cells were exposed to two 10-min treatments with fluconazole or caspofungin. No change in Ca^2+^-GCaMP6 spiking was seen, and there was no rise in non-spiking Ca^2+^-GCaMP6 signals (Fig. S7). The lack of an immediate stress response to these antifungal compounds during either the first or second exposure suggests that their effects are not sensed by cells within this time frame. This suggests that, unlike the chemical attack on global cell integrity made by SDS or H_2_O_2_, inhibition of ergosterol or β-glucan biosynthesis by antifungal drugs does not trigger an immediate stress response, which may be delayed until molecular depletion is sensed in growing cells. Alternatively, it is possible that Ca^2+^-entry was already at sufficient levels to function in a stress response, such that influx rates were not affected.

## DISCUSSION

### The use of genetically encoded Ca^2+^ reporters in fungi

Despite the importance of Ca^2+^-signaling and homeostasis in fungal pathogens, our understanding of real-time Ca^2+^-dynamics in single cells has been limited by the paucity of effective reporters in fungi. The development of new genetically encoded [Ca^2+^] indicators has allowed examination of the role of Ca^2+^ flux in the growth of filamentous fungi in real time. The Cameleon Förster resonance energy transfer (FRET) ratiometric system used Ca^2+^-binding to calmodulin and the M13 calmodulin-binding peptide to transfer energy from CFP to YFP to visualize cytosolic Ca^2+^ (33, 34). The Cameleon reporter was expressed in the filamentous fungi, *Fusarium oxysporum, F. graminearum,* and *Magnaporthe oryzae,* where real-time imaging revealed pulsatile changes of ~26 s in duration in apical Ca^2+^ during hyphal growth for the first time. Tip-focussed Ca^2+^ spiking was observed in *Colletrotrichum graminicola* using the same reporter although spiking did not correlate with the non-pulsatile tip growth rate. More recently, Ca^2+^-reporters have been developed from GCaMP, a construct using the Ca^2+^-calmodulin-M13 peptide interaction to excite circularly permutated GFP (35 – 37). R-GECO was a GECI derived from GCaMP3, the third iteration of GCaMP, and its visualization in *Aspergillus nidulans* and *Neurospora crassa* led to a model of oscillatory Ca^2+^ entry followed by actin disassembly and vesicle exocytosis, a cycle that correlated with the step-wise nature of hyphal tip extension in these fungi (36). GCaMP6 has been adapted for expression in *A. fumigatus*, where heterogeneous Ca^2+^ waves were imaged in hyphae and germlings and dynamics were altered by exposure to high (200 mM) Ca^2+^ (38). It has also been used in *Schizosaccharomyces pombe*, where transients during cell-separation events, hypo-osmotic and Ca^2+^ shock were observed (39). Here, we engineered a codon-optimized version of GCaMP6f in order to visualize Ca^2+^ dynamics in the yeast form of the fungal pathogen, *C. albicans*, for the first time and dissect their role in stress responses.

### Analysis and model for Ca^2+^-GCaMP outputs in *C. albicans*

**Fig 7:**
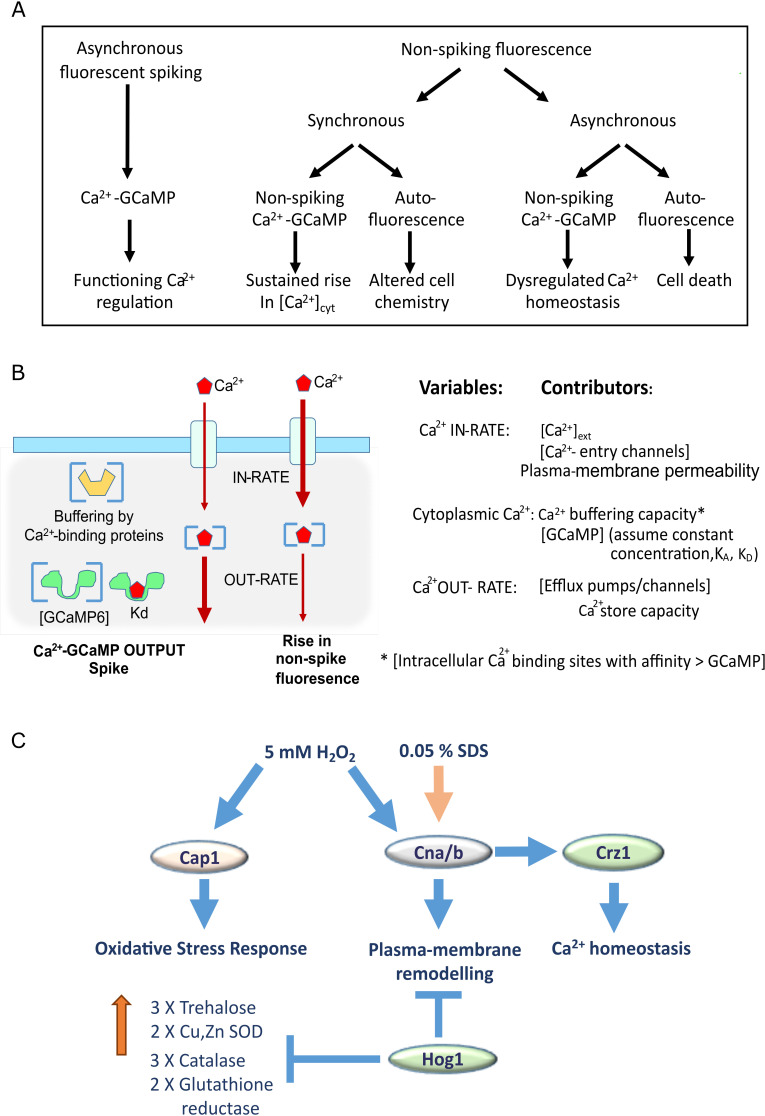
(**A**) Categorization of fluorescence outputs from GCaMP imaging in *C. albicans*. See text for details. (**B**) Proposed mechanisms for how Ca^2+^ influx and efflux rates are affected by variables but together deliver spiking vs non-spiking Ca^2+^ GCaMP signals. (**C**) Model for adaptation to oxidative and membrane stress in *C. albicans*. Oxidative stress requires Cap1 and calcineurin signaling for upregulation of antioxidant enzymes and remodeling of the plasma-membrane. Ca^2+^ GCaMP spiking is not inhibited in the *hog1∆* mutant as it is pre-adapted through release of gene-expression inhibition, which may reduce plasma-membrane permeability to H_2_O_2_. Calcineurin is necessary for adaptation and responses to both H_2_O_2_ and SDS, also via plasma-membrane remodeling. Crz1 is required for maintenance of Ca^2+^ homeostasis during stress responses.

The imaging output from our perfused microfluidics system showed three variants of fluorescence output from GCaMP-expressing cells, two of which split further on comparison with the output from the non-GCaMP strain (transformed with an empty-vector) to give five informative outputs overall (Fig. 7A).

1. Asynchronous Ca^2+^-GCaMP spiking in individual cells was observed in both resting cells and on application of a stress treatment. That spiking was never completely synchronized across the cell population suggests that the ability of a cell to produce a Ca^2+^ spike may depend on an intrinsic cell state. Cells undergoing Ca^2+^-GCaMP spiking were assumed to be viable and have a functioning Ca^2+^-regulatory system. Cells that stopped spiking, for example in the presence of 1 M NaCl, were able to re-start on removal of the stress so lack of spiking was not indicative of cell death. Imaging in the millisecond range showed that spike duration was 5–6 s, with a time to peak of 1–2 min, much slower than the tens-of-milliseconds for GCMP6f in neuronal cells, but much faster than the 26 s transients reported in hyphal filaments (10). However, due to the onset of photo-stress, the millisecond frame rate could not be sustained for the 30 min period required to characterize each stress exposure in our system. Imaging every 5 s, therefore, satisfactorily captured spike rates while minimizing photo-stress during the period of interest (Fig. S2). However, this rate did not allow examination of inter-spike intervals in single cells, which are likely to have an interval minimum that may vary according to Ca^2+^-channel number, regulation, and environmental conditions.
2. Synchronous non-spiking fluorescence across the population occurred in response to exposure to high [Ca^2+^]_ext_, H_2_O_2_, the first treatment with SDS and on washout of 1 M NaCl. While the intensity of these signals varied by cell, they followed the same overall magnitude and time-course trend across the population. Comparison of the fluorescence output between cells expressing GCaMP vs a strain transformed with an empty vector allowed us to differentiate further between non-spiking Ca^2+^-GCaMP-derived signals and autofluorescence. The only incidences that we observed for synchronous non-spiking Ca^2+^-GCaMP signals were during the first few minutes of exposure to SDS and at the commencement of washout of 1 M NaCl, where the absence of these signals in the empty-vector strain indicated that they were Ca^2+^-GCaMP-dependent. Synchronous non-spiking fluorescence that was seen in both strains (e.g., during exposure to H_2_O_2_) were considered to be autofluoresence. As neither of these whole-population responses resulted in loss of cell viability (Fig. S3), we concluded that synchronous rises in non-spiking Ca^2+^-GCaMP signals were due to a sustained rise in [Ca^2^]_cyt_ (Fig. 7B and model below), while synchronous rises in autofluorescence during treatment phases were due to altered intracellular chemistry, particularly during exposure to H_2_O_2_.
3. Asynchronous non-spiking fluorescence was seen in a sub-population of cells on treatment with SDS or H_2_O_2._ In SDS, this was due to sustained autofluorescence as it was observed in cells transformed with either GCaMP or the empty vector. It occurred in a large number of cells, commencing during the last minutes of SDS exposure and continuing through the stage 3 washout period. Propidium iodide staining after the third exposure to SDS and tracking the output from each PI-positive cell back through the time-course showed a positive correlation between a cell’s autofluorescence and its subsequent death. The sub-population of cells that did not autofluoresce survived all three treatments with SDS. The asynchronous autofluorescence signal in the *cnb1*Δ mutant was additionally informative because it revealed that all cells had died on the second exposure to SDS (Fig. 3D). In contrast, the asynchronous fluorescence seen after H_2_O_2_ exposure occurred only in GCaMP-expressing strains and not in the empty-vector strain. The signal was, therefore, Ca^2+^-GCaMP dependent. These signals rose and fell erratically in individual cells and appeared to be generated by dysregulated Ca^2+^ homeostasis. However, this did not lead to cell death, unlike the sustained autofluorescence seen in SDS.

The cellular mechanisms underpinning the generation of spiking vs non-spiking Ca^2+^-GCaMP signals require further exploration but may simply depend on the ratio of cytoplasmic [Ca^2+^] to Ca^2+^-GCaMP binding sites, which, in turn, is defined by the in rate vs the out rate of Ca^2+^ flux (Fig. 7B). Our model proposes that if the out rate (i.e., the removal of cytoplasmic Ca^2+^ via plasma-membrane pumps or into intracellular stores) is greater than the in rate, then any Ca^2+^ ions entering the cell that that bind GCaMP will, on dissociation, be quickly removed from the cytoplasm. Conversely, if the in rate is greater than the out rate, then [Ca^2+^]_cyt_ will increase and dissociated Ca^2+^ will remain in the cytoplasm to repeatedly re-bind to GCaMP, thus yielding a sustained rise in fluorescence. The many potential variables that may affect this balance include buffering by indigenous Ca^2+^-binding sites, the status of intracellular stores and the function of Ca^2+^-channel regulators, all of which may be altered by changes in the intracellular biochemistry wrought by stressors and by adaptive gene expression. The effect of these variables on Ca^2+^-GCaMP dynamics remains to be investigated.

### Adaptation to SDS and H_2_O_2_ is likely due to plasma-membrane remodeling

Unlike the consistent shutdown of Ca^2+^-GCaMP spiking seen in high NaCl, cells treated repeatedly with SDS or H_2_O_2_ (with a 20-min recovery period between each exposure) exhibited an ability to adapt, whereby the third application of the stress no longer inhibited spiking. In the first treatment with SDS, a detergent that disrupts membrane integrity, the spike-burst was followed by a population-wide rise in the non-spiking Ca^2+^-GCaMP signal, suggesting that partial disruption of the plasma-membrane allowed a sudden influx of Ca^2+^ into the cytoplasm that temporarily overwhelmed the homeostasis mechanisms. Around 80% of cells did not survive this first exposure and showed rises in autofluorescence that declined slowly over 20 min or more, consistent with leakage of cytoplasm from damaged membranes. Cell death was due to Ca^2+^ overload as inclusion of 10 mM BAPTA with SDS treatment led to almost 100% survival (Fig. S3). The surviving 20% of cells were able to generate Ca^2+^ spikes during the second and third exposures and did not display further rises in non-spiking Ca^2+^-GCaMP signals, maintaining viability to the end of the experiment. The first exposure to SDS may have selected a sub-population that was already partially resistant to SDS. The finding that the *cnb1*Δ mutant was unable to survive in SDS suggests that resistance to SDS in the presence of extracellular Ca^2+^ is mediated by calcineurin and is likely to involve properties of the plasma-membrane.

Calcineurin and the oxidative-stress-related transcription factor, Cap1, were both required for restoring Ca^2+^-GCaMP spiking and synchronous, non-lethal rises in autofluorescence in 5 mM H_2_O_2_. Cap1 activity has been well-characterized in terms of its role in up-regulating antioxidant enzymes for neutralizing H_2_O_2_ as a key resistance mechanism in the oxidative stress response (24, 40). The finding that the *hog1*Δ mutant was pre-adapted is consistent with its increased trehalose content and up-regulated catalase A, which are expected to provide intracellular protection against H_2_O_2_ (20, 41). However, in *S. cerevisiae*, no correlation was found between the capacity to neutralize cytoplasmic H_2_O_2_ and resistance (42). Instead, during exponential-growth, resistance to H_2_O_2_ was conferred by a twofold decrease in plasma-membrane permeability through membrane remodeling (42, 43). The downstream effector of Hog1 in the oxidative stress response is not known (26), but this study suggests that it is involved in cross-talk with calcineurin signaling and may act by repressing genes involved in membrane remodeling that ultimately controls permeability to H_2_O_2_ (Fig. 7C).

### Conclusion

The genetically encoded GCaMP Ca^2+^-reporter has enabled us to identify five different fluorescence outputs from *C. albicans* cells in response to a number of commonly applied stressors. The outputs are highly informative but reveal new levels of complexity that require integration into existing cell response models. Here, we focused on results observed for treatments that immediately elicit a stress response in the cell. Optimization of imaging regimens will be required in order to capture delayed or slower responses, including exposure to antifungal drugs that target specific biosynthesis pathways, without inducing photo-stress. Nevertheless, this study opens the door to the use of the GCaMP reporter to further probe these and other cell stress responses and to dissect the role of Ca^2+^-homeostasis and signaling in stress adaptation mechanisms.

## MATERIALS AND METHODS

### Generation of GCaMP6-expressing *C. albicans* strains

The strains and plasmids used in this study are shown in Tables S1 and S2. G-CaMP6f (10) was synthesized by GeneArt, Germany, incorporating modifications for *C. albicans* by circular permutation of *yEGFP3* (12, 44) and codon optimization of the Ca^2+^ Calmodulin and M13 domains (Fig. S1). Plasmids and sequences are available at https://doi.org/10.5281/zenodo.8009704. GCaMP6 was subcloned between the *Age*I and *Sph*I sites in plasmid CIp-*NATflip-pACT1-ScCyct* (45, 46) to generate plasmid B153. Plasmid B251 bearing *URA3* as the selectable marker was generated by cloning *ACT1p-GCaMP6* into the *Mlu*I and *Kpn*I sites of the CIp10 plasmid (47, 48). Plasmid B250, without GCaMP6, was transformed into cells as the empty-vector strain. Plasmids were linearized with *Stu*I. B153 and B250 were transformed into SC5314 and B251 was transformed into the Ura-minus strain, DSY3891, to generate strains A567, A569, and A585, respectively. Transformants were selected on solid yeast-Bacto-peptone-dextrose or Sabouraud-dextrose plates containing 200 µg/mL nourseothricin or 0.67% yeast-nitrogen base without supplements and confirmed by restriction digest, fluorescence microscopy, and PCR using primers NATR fw 5-AGCTTGTTCACCATCGGAAG-3 and NATR rv 5-TTCTGTTCCAGGTGATGCTG-3 or primers SP RPSgs rv 5-GGTAGTCGATATTCAGGGCC-3 and GCaMP6-1R 5-CCTTCAAACTTGACTTCAGC-3.

### Imaging of Ca^2+^-GCaMP responses in yeast

Cells were grown overnight at 30°C in SD (0.67% [wt/vol] yeast nitrogen base with amino acids and ammonium sulfate) medium with shaking at 200 rpm. Approximately 2 × 10^6^ cells in 200 µL were washed three times with ddH_2_O and re-suspended in 75 µL modified Soll’s medium (MSM) (49) ± Ca^2+^ before imaging at 30°C in Y04C microfluidic plates (Millipore Merck, UK) connected to an Onix microfluidic perfusion system (CellASIC Corp., USA). Prior to cell loading, the PBS from two wells and the cell well was flushed through with 75 µL medium by sealing the plate and applying 2 × 6 psi for 10 s, followed by 2 min at 5 psi from each medium well. The remaining medium was removed and 300 µL medium corresponding to the two experimental conditions (control and shock) was added to each well. Pressure (3 psi) was applied to the “shock” well for 1 min to reduce the lag time between pressure application and medium entering the cell well, as determined by calibration experiments using a fluorescent dye. Cells (75 µL) were loaded into the cell well with a ceiling height of 3.5 µm where 1–2 pulses at 6 psi for 10 s yielded ~100 cells/field of view. MSM was perfused at 2 psi. A DeltaVision Core microscope (Image Solutions Ltd., UK) with a CoolSNAP camera (Photometrics United Kingdom Ltd., UK), a 60× objective, and a GFP filter was used for live-cell imaging. After plate loading, cells were incubated for 40 min in “baseline” conditions. At *t* = 40 min, stage 1 imaging in DIC and GFP commenced at position 1 at 5 s intervals for 5 min, with an auto-focus step every 2 min. At stage 2, *t* = 45 min, cells were exposed to a stress compound for 10 min before stage 3, a return to baseline conditions with continued imaging to *t* = 70 min. After a 5-min interval, imaging and treatment (exposures 2 and 3) were repeated at microscope positions 2 and 3, and without treatment at position 4 as a control to confirm long-term cell viability. Alternatively, to assess cell death following three treatments, propidium iodide (1 µg/mL) was perfused into the chamber and cell staining imaged and quantified. To assess imaging artifacts, cells expressing the empty-vector were imaged, and GCaMP cells were imaged in baseline (stage 1) conditions of 5 mM Ca^2+^ with a switch of perfusion channels but with no treatment. Baseline conditions in all experiments were set at 5 mM Ca^2+^ and pH 7.5 (to maintain pH ≥ 7 in the presence of added reagents) unless otherwise stated. To determine GCaMP spike shape, CAI4-GCaMP was grown in MSM pH 7.5, 40 mM CaCl_2_ in an Ibidi U slide (Thistle Scientific, UK) and imaged on a Dragonfly spinning disk confocal microscope (Oxford Instruments, UK) at a frame rate of 146 ms.

### Imaging of Cap1-GFP localization

Cap1-GFP cells were grown overnight at 30°C in YPD (2% mycological peptone [wt/vol], 2% glucose [wt/vol], 1% [wt/vol] yeast extract) with shaking at 200 rpm. Cells were harvested, washed three times in ddH_2_O, and resuspended to ~1 × 10^6^ cells/mL. Cells (100 µL) were added to an Ibidi U slide, allowed to adhere, and incubated in MSM + 5 mM H_2_O_2_ for 15 min at 30°C. Cells were imaged in DIC and FITC every minute before the addition of 5 mM H_2_O_2,_ and at every minute thereafter for 15 min.

### Image analysis software

Two-channel image sequences were imported into MatLab using the BioFormats package (50). After correction for stage drift, if necessary, image series were aligned to the first time-point by (*x,y*) translation estimated using the MatLab imregtform function and applied using the imwarp function. Aligned images were filtered with a 5 × 5 (*x,y*) pixel box average to reduce noise, sub-sampled by a factor of 3 to decrease processing time by removing redundant information after smoothing, and normalized in the range [0 1]. The average background intensity was measured from a manually defined region of interest (ROI) for each time-point and subtracted.

As cells did not move during the time-series, an essentially noise-free template image was constructed using additional Gaussian smoothing in (*x,y*), with σ set to the minimum cell radius (typically four pixels), followed by an average projection of the time-series in *t*. The initial segmentation used a mid-grey local adaptive filter, which uses a spatially varying threshold set as the mean after erosion and dilation with circular neighborhood set by the maximum cell radius (typically 25 pixels). Touching cells were separated using a watershed transform of the Euclidean Distance Transform of the binarized cells, with additional suppression of maxima less than 15% of the maximum to avoid over-segmentation. Within each cell territory, the actual cell boundary was defined as 50% of the local intensity maximum in each territory. Cells touching the border or below a minimum area (typically ~40 pixels set by the minimum cell radius) were excluded from further analysis. Each ROI was given a unique label.

A 50-s photobleaching period was observed within the first 10 frames of fluorescence imaging and was excluded from analyses. As the signal from each cell declined due to photobleaching, the baseline fluorescence (*F_b_*) was estimated using a rolling median filter with a large window size (typically 60 frames or 5 min) to exclude spikes or transients. In cases where there was an extended shift in the saturation Ca^2+^ level, e.g., during H_2_O_2_ treatment, the baseline was calculated using the MatLab msbackadj function, which finds points from the lowest 10% quantile in multiple shifted windows (typically 60–120 frames or 5–10 min duration), with a typical step size of 30 frames (150 s). Local baseline values were then fit using loess (quadratic) regression.

The *δF/F_b_* ratio and the fold change in fluorescence, *F/F_b_*, were calculated from the background-subtracted signal and the baseline. Spikes and changes in resting level were separated using a rolling median filter with a typical window size of 7 frames (35 s). The first 10–20 frames (50–100 s) were excluded to avoid the initial peak in signal following perturbation during experiment set-up. Changes in non-spiking GCaMP levels were calculated as the fold change above baseline, after the median filter. Spikes were identified from the ratio between the background-subtracted value and the median filtered data using the MatLab findpeaks function. Spikes were defined as a signal fold change of >1.5, a prominence (difference to the next peak) >0.2, and the maximum width <10 frames (50 s). The number of spikes/cell was determined, along with the fold-change and width (dependent on the sampling rate, during each stage of the experiment. If there was more than one spike in a cell, the inter-spike interval was determined. The overall spike frequency was calculated as spikes cell^−1^ min^−1^ to normalize between different experiments with varying number of cells and treatment duration. Data were output to Excel. The background effect of photo-stress was quantified using control experiments but found to be insignificant during the first 20 min of imaging. Falsely identified cells, due to background heterogeneities, were manually removed.

A pseudo-color-coded image of the Ca^2+^ transients was constructed by assigning the *F/F_b_* ratio value for each cell to the corresponding ROI, imposing display limits of [0.8 3], and converting to a rainbow color scale running from blue to red. The coded ratio was then superimposed on the bright-field image. The software (V1.05.00) and user manual are freely available for download from https://doi.org/10.5281/zenodo.8064064. Future updated versions will be accessible from https://doi.org/10.5281/zenodo.8064064. Plots of spiking and non-spiking GCaMP activity were generated using a bespoke MATLAB script, which imported processed data generated from the previous analysis.

Changes in the non-spiking level of Ca^2+^-GCaMP signal on exposure to stress were differentiated from changes in autofluorescence by comparing the signals between GCaMP-expressing strains and strains transformed with the empty GCaMP vector as a control. To determine changes in cell volume, cells were first segmented in the DIC channel using the Yeast Spotter segmentation software (Lu et al., 2019) in Python V3.6, using the PyCharm IDE (2021.2.3). Separated images were placed into an input directory, and the pre-defined weights for the neural network were downloaded from https://zenodo.org/record/3598690/ and placed into the same directory as the segmentation and opts.py files. The segmentation script was run resulting in FIJI compatible masks. The cells were assumed to be spheres, and the volume was calculated from the radius and area of each segmented cell. The area of biological replicates was averaged and normalized to the initial volume.

### Data analysis

In Excel, spike values were displayed in a chronological order, allowing separation into stages 1–3 to identify trends. The number of spikes/cell/min was quantified for baseline (stage 1), 10 min of stress treatment (stage 2) and 15 of washout (stage 3). In stage 2, spikes/cell/min were calculated in a 5-min window commencing 2.5 min after wash-in and ending 2.5 min before stage 3 washout. Spike rates generated by increasing [Ca^2+^]_ext_ were analyzed using a one-way ANOVA and a post-hoc Dunnett’s multiple comparisons *t*-test.

The percentage of cells spiking in each field of view during these intervals was calculated. To compensate for variability between Ca^2+^-increase experiments, spiking was normalized with respect to the first 4 min of control. The first 10 frames were discarded due to rapid initial photo-bleaching. “Adaptation factor” to H_2_O_2_ was determined by quantifying spikes/cell/min for each exposure 1, 2, and 3 (using the 5 min window) and determining the gradient (adaptation factor) of the line of best fit across the exposures.

Ca^2+^-GCaMP output from Dragonfly confocal imaging was analyzed with a bespoke MATLAB script. Individual cells of interest were cropped by hand and thresholded so that pixels below a minimum intensity were replaced with black, those above a maximum intensity were replaced with white, and linear interpolation was used for pixels with intermediated intensities. Photo-bleaching was corrected by multiplying each pixel intensity by a 1 + *αt*, where *α* = 3.4 × 10^−3^ s^−1^ and *t* is the time in seconds. For any given time point, the Ca^2+^-GCaMP signal was defined as the mean intensity within each cell. Traces were smoothed using the MATLAB smoothdata() function, before being scaled so that all spikes lie between 0 and 1.

## SUPPLEMENTAL MATERIAL

Supplemental material mbio.01157-23-s0001.docx Fig. S1-S7; Tables S1 and S2.

ASM does not own the copyrights to Supplemental Material that may be linked to, or accessed through, an article. The authors have granted ASM a non-exclusive, world-wide license to publish the Supplemental Material files. Please contact the corresponding author directly for reuse.
