# Supplementary material for: Dynamic calcium-mediated stress response and recovery signatures in the fungal pathogen, Candida albicans
Source: mBio. 2023 Sep 26;14(5):e01157-23. doi: 10.1128/mbio.01157-23 (PMC10653887; doi:10.1128/mbio.01157-23)
Supplement: Supplemental material — Fig. S1-S7; Tables S1 and S2. [file mbio.01157-23-s0001.docx]

**Dynamic calcium-mediated stress response and recovery signatures in the fungal pathogen, *Candida albicans***

Giuraniuc CV^4^*, Parkin C^1^*, Almeida MC^4^, Fricker M^3^, Shadmani P^2^, Nye S^2^, Wehmeier S^4,^ Chawla S^4^, Bedekovic T^1,4^, Lehtovirta-Morley L^4^, Richards D^2,5^, Gow NA^1,4^ and Brand AC^1,2,4‡^

*Joint first authors

^1^MRC Centre for Medical Mycology at the University of Exeter, Exeter, UK

^2^Living Systems Institute, University of Exeter, UK

^3^ School of Plant Sciences, University of Oxford, Oxford UK

^4^School of Medicine, Medical Sciences & Nutrition, University of Aberdeen, UK

^5^Department of Physics and Astronomy, University of Exeter, UK

^‡^Corresponding author: a.brand@exeter.ac.uk

**Supplementary Material**

**Supplementary movies:**  [<https://doi.org/10.5281/zenodo.8179089>]


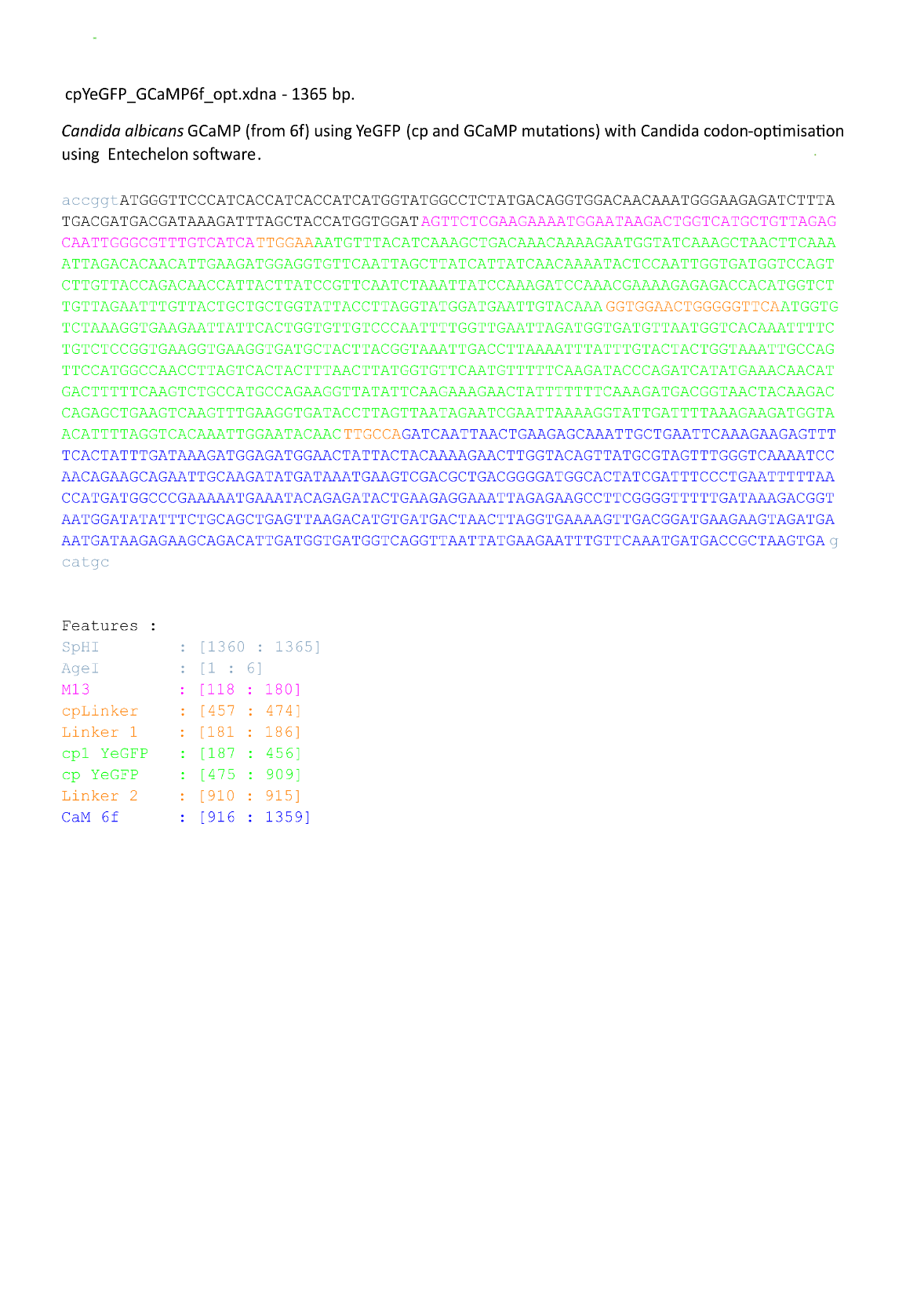

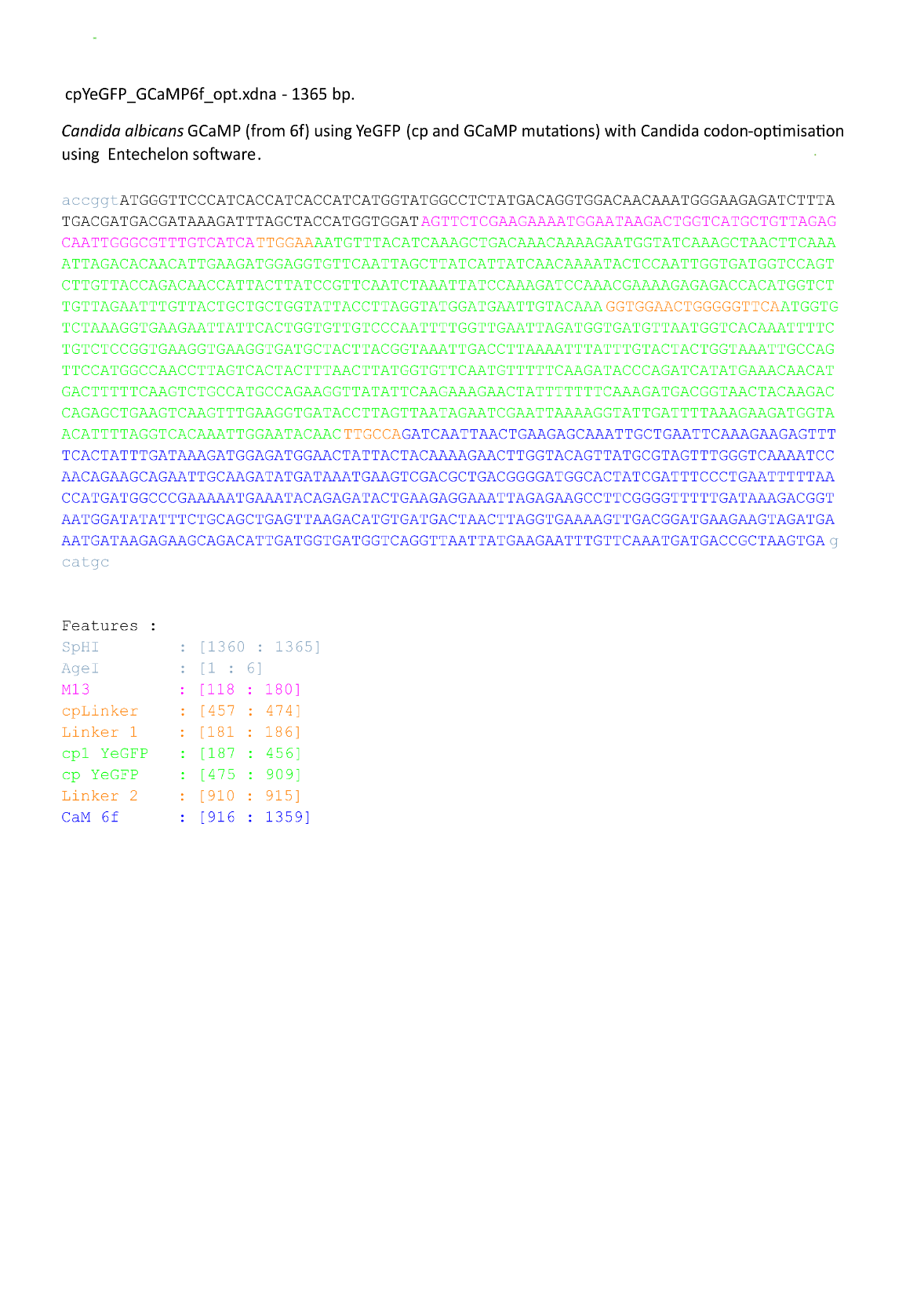


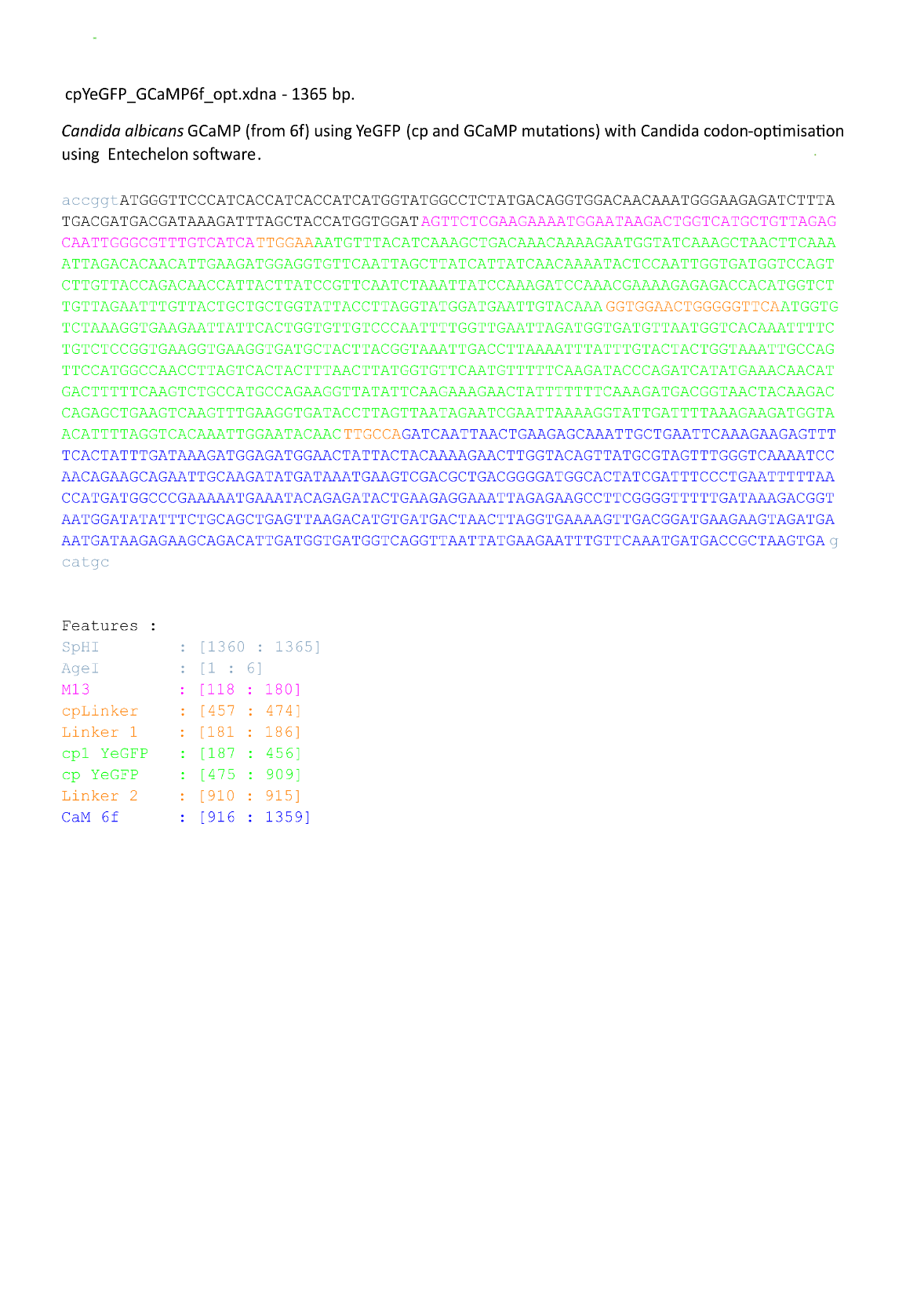


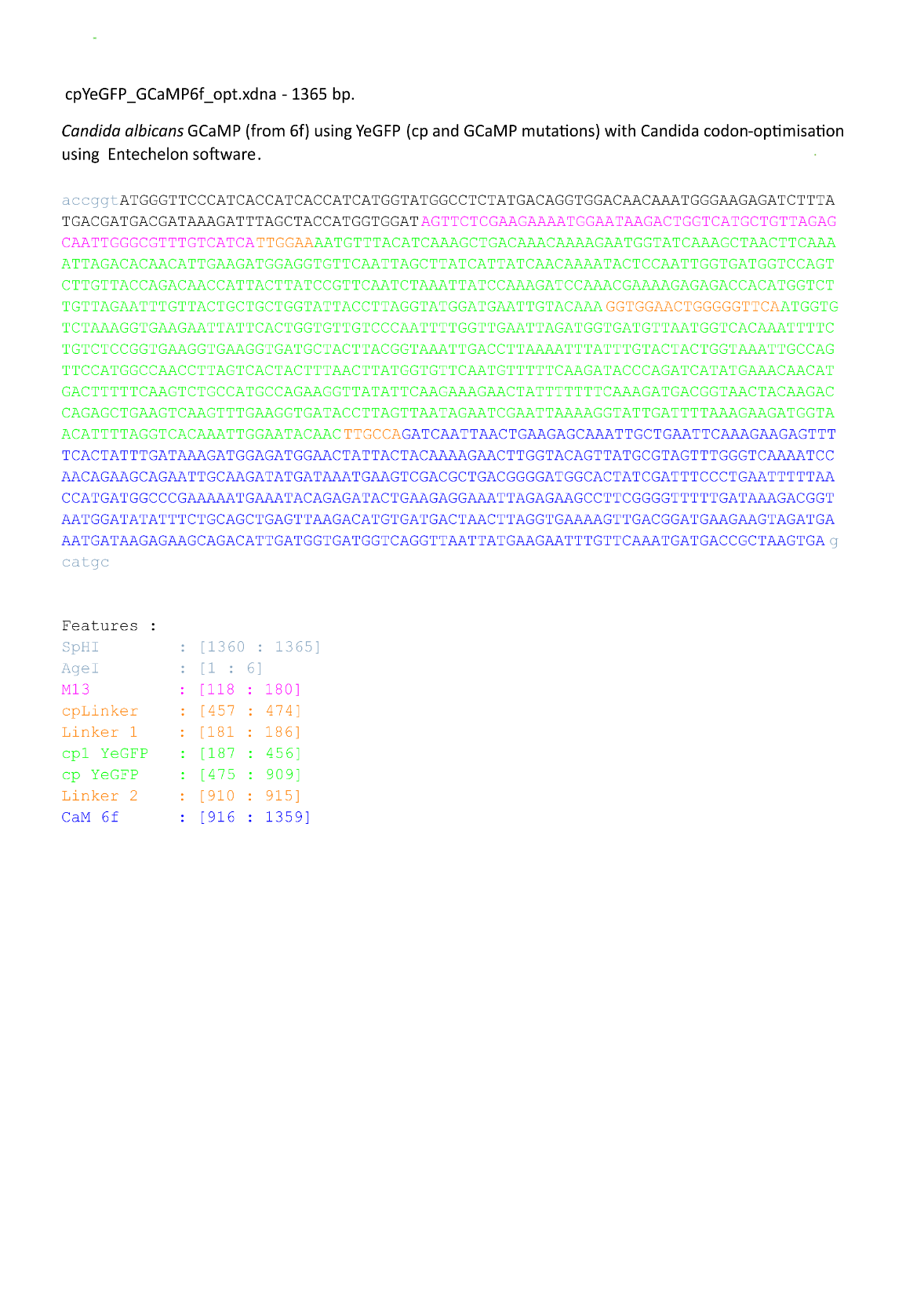


**Fig. S1: Codon optimised nucleotide sequence of GCaMP6f for expression in *C. albicans*.** GCaMP6f coding sequence was taken from Chen et al., 2013, codon optimised for expression in *C. albicans* and synthesised in plasmid Ca-GCaMP6f by Geneart. Features of coding sequence are highlighted.

**Table S1: *C. albicans* strains used in this study**

**
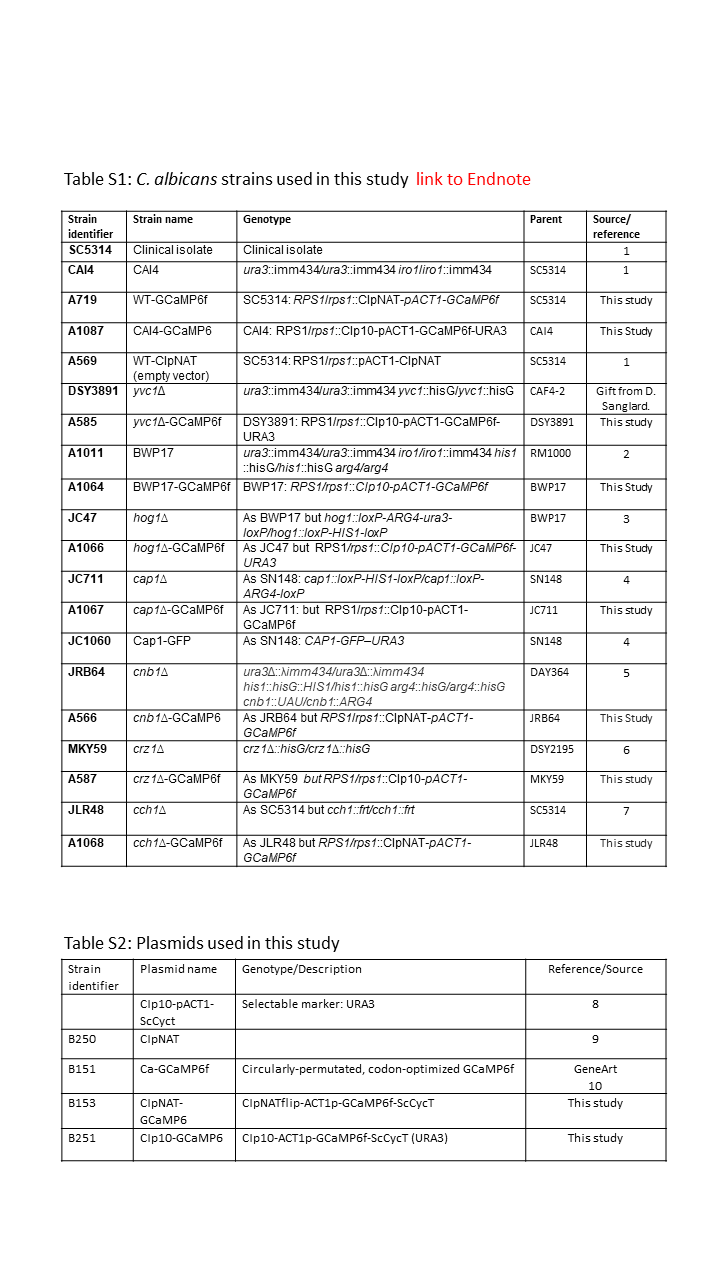
**

**Table S2: Plasmids used in this study**

**
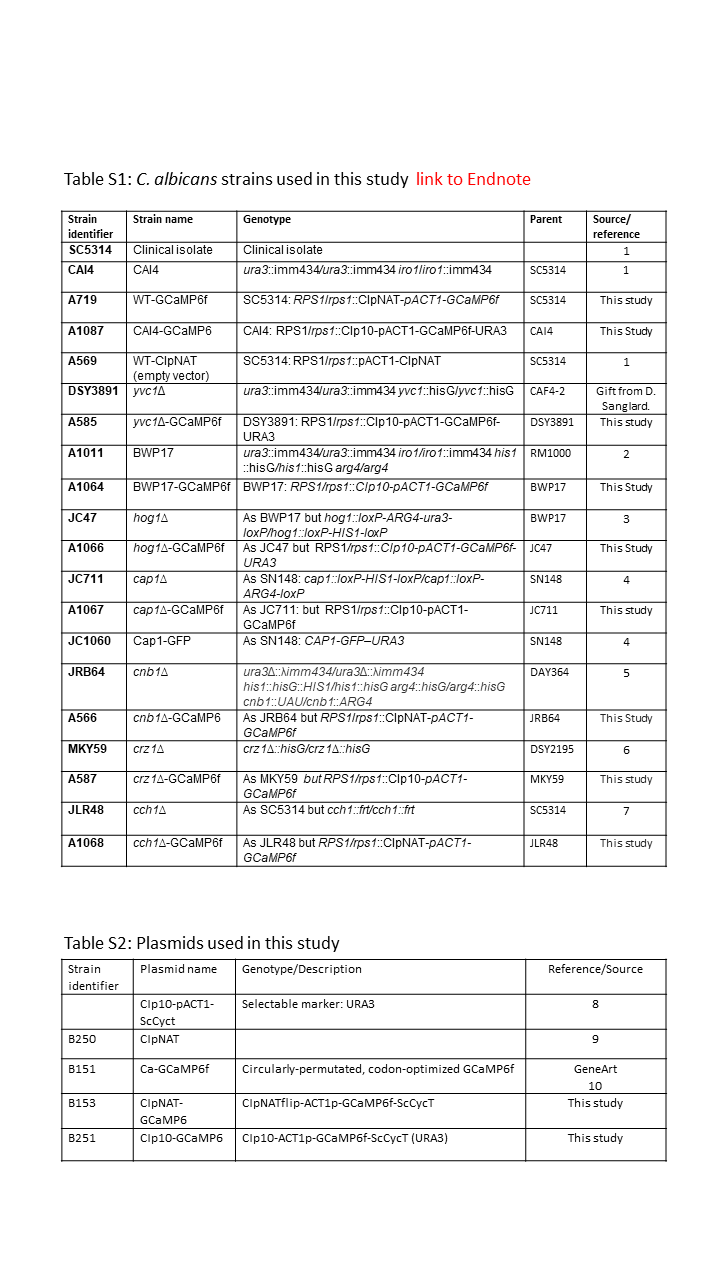
**


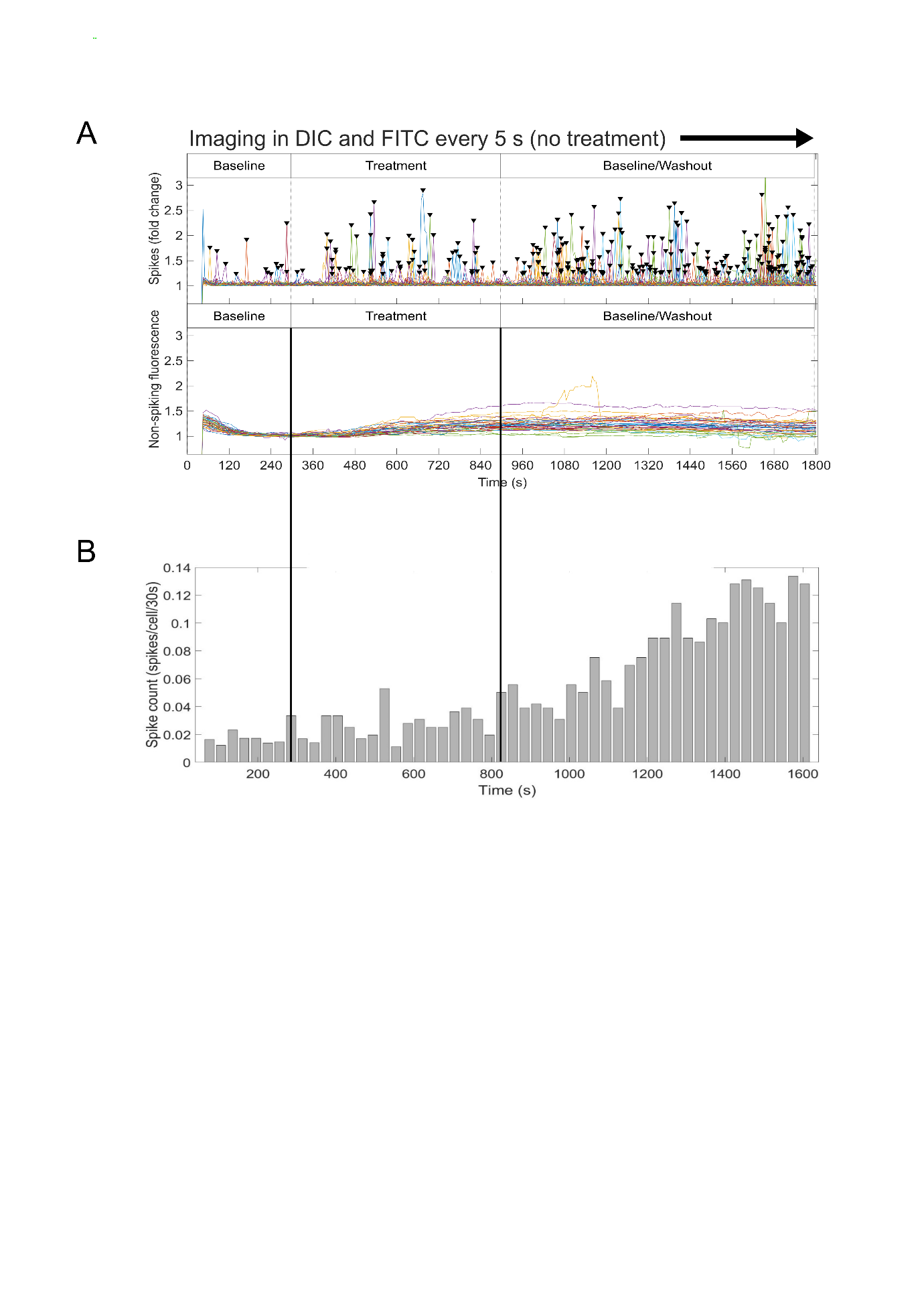


**Fig. S2: Onset of photo-stress occurs towards the end of Stage 3 (washout) of the experimental time-course.**

A. Representative Ca^2+^-GCaMP plots of the wild-type strain, BWP17-GCaMP, in the presence of 5 mM Ca^2+^  (pH 7.5) throughout the entire experimental time course, with no imposed treatment. B. Spikes/cell/30 s window were plotted over the same time course.

**
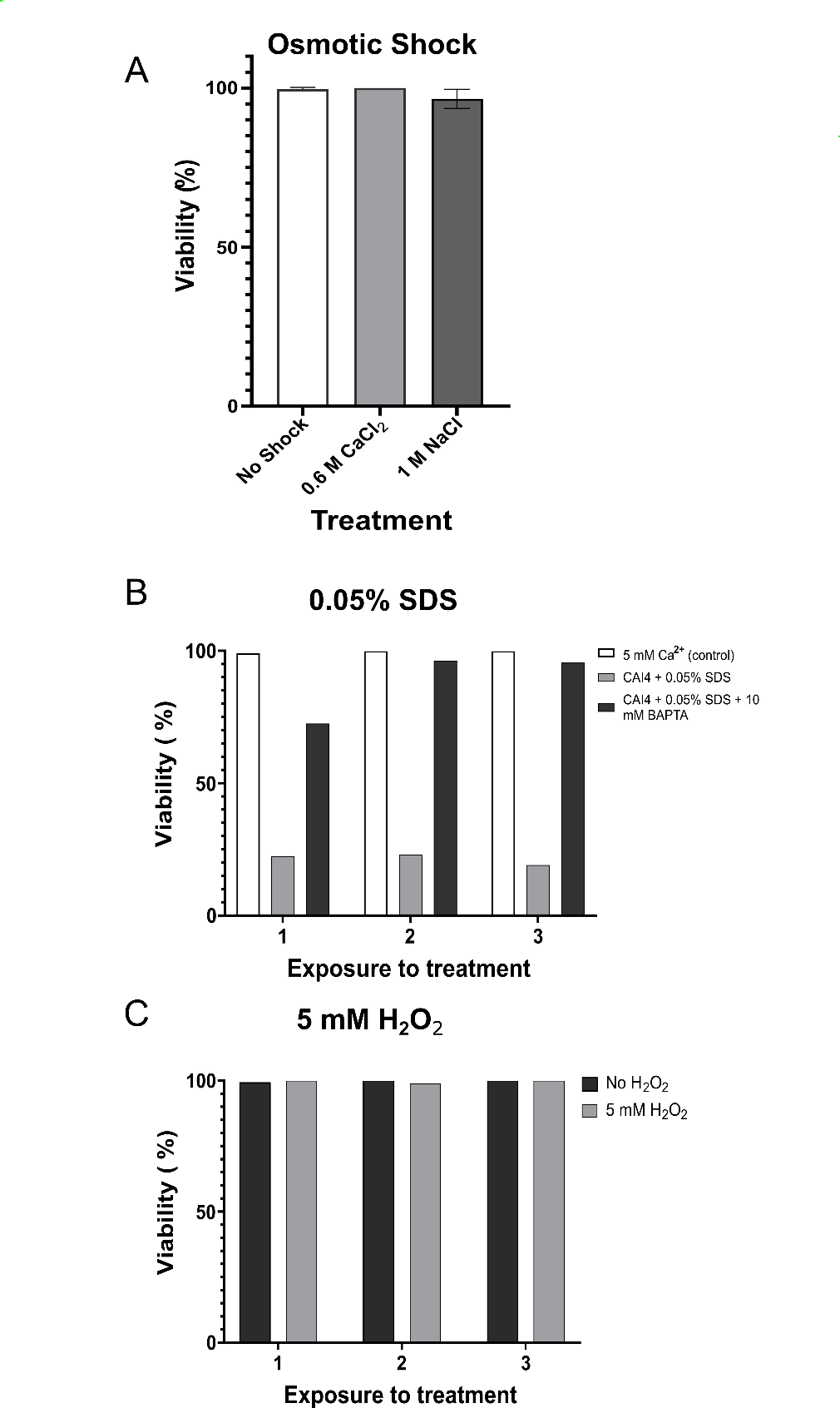
**

**Fig. S3: Cell viability after 3 exposures to osmotic shock, SDS, or H_2_O_2_** A. The empty vector strain (A569) underwent 3 exposures to either 0.666 M CaCl_2_ or 1 M NaCl (in 5 mM Ca^2+^) followed by staining with 1 ug/ml PI at the end of E3. % viability was determined as the percentage of non-stained cells in the population. The no-shock control was BWP17-GCaMP in the presence of 5 mM Ca^2+^ and viability was determined as above. Bars = mean ± SD across 3 exposures. B. In 5 mM Ca^2+^, CAI4-GCaMP was exposed 3 times to 0.05 % SDS or 0.05 % SDS + 10 mM BAPTA in S2 of the experimental time course, and % viability was calculated as in (A). C. BWP17-GCaMP was exposed 3 times to 5 mM H_2_O_2_ (in 5 mM Ca^2+^) during S2 of the experimental time course and % viability was calculated as in (A).


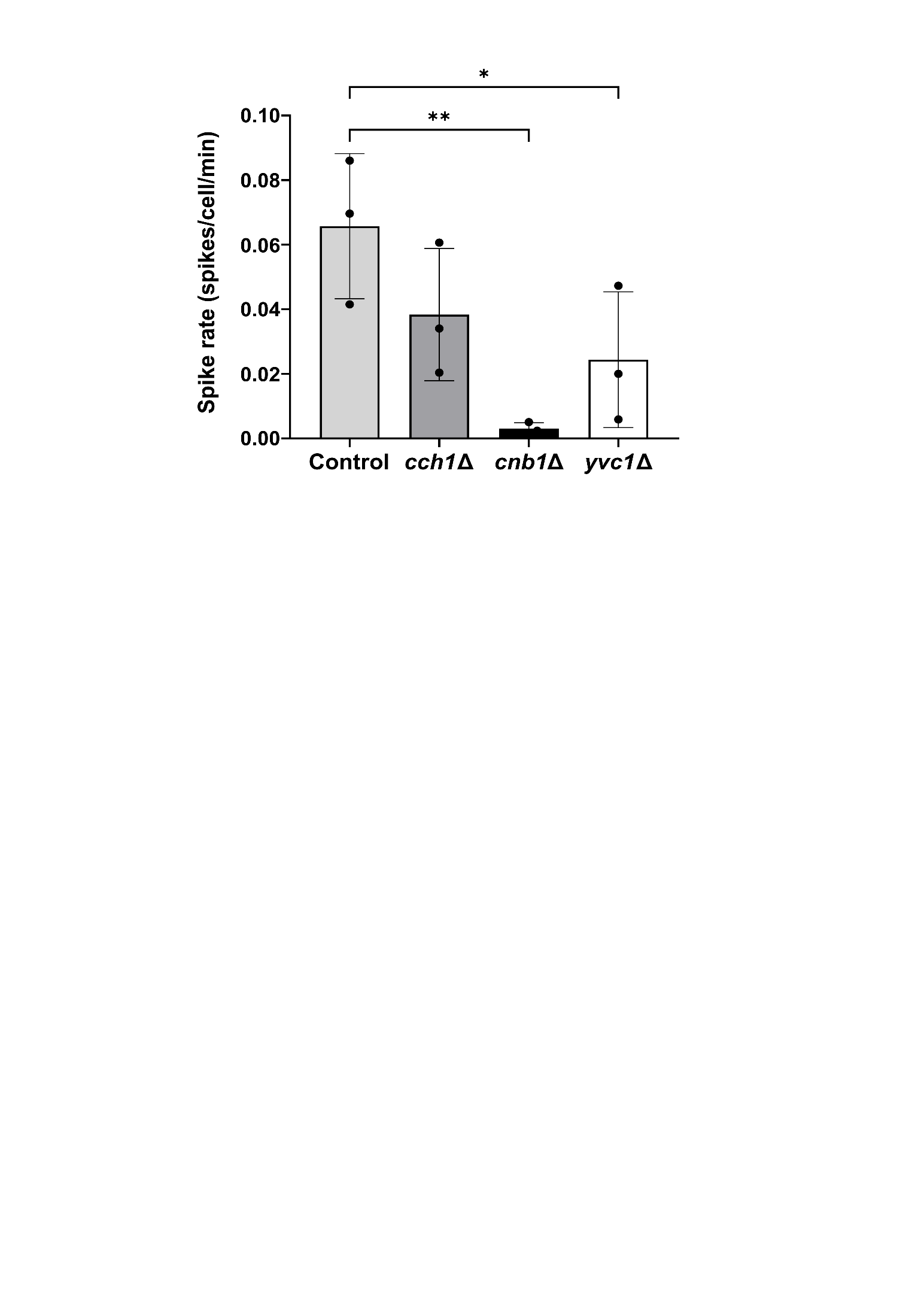


**Fig. S4: Comparative spike rates for wild-type-GCaMP and gene-deletion strains**

Spike rates (spikes/cell/min) in resting *cch1*Δ, *cnb1*Δ and *yvc1*Δ mutants expressing GCaMP were determined during Stage 1 at the first microscope position (ie, prior to exposure to any treatment compound) and compared with the wild-type-GCaMP strain using a one-way ANOVA with Dunnett’s *post hoc t*-test. * = *p* ≤0.05, ** = *p* ≤ 0.01.


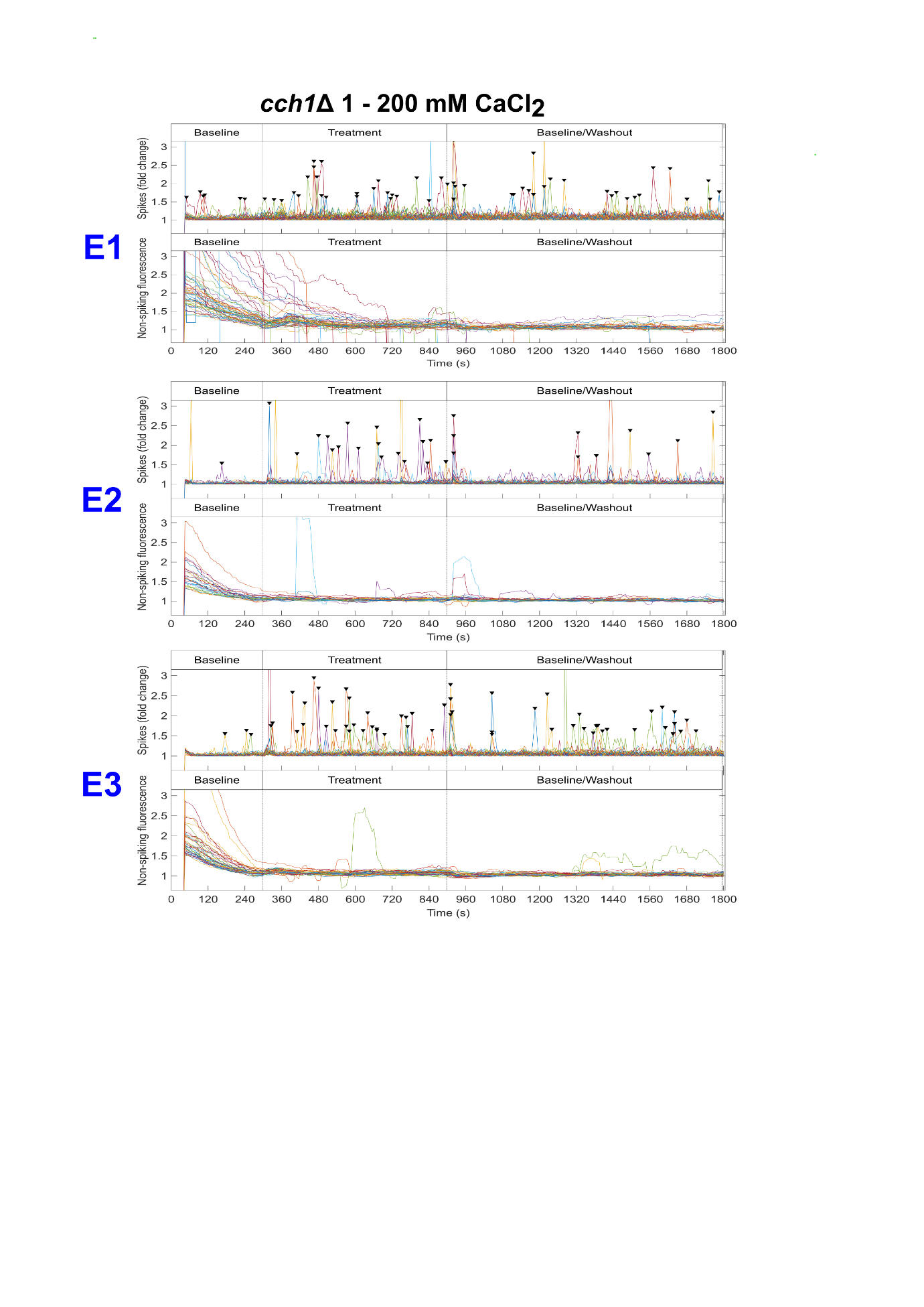


**Fig. S5: Deletion of Cch1 does not abolish GCaMP-spiking and *cch1Δ*-GCaMP remains responsive to changes in [Ca^2+^]_ext_**

*cch1∆*-GCaMP was acclimated to 1 mM Ca^2+^ during S1 and exposed to 200 mM Ca^2+^ during S2 before reverting to 1 mM Ca^2+^ in S3 (E1). This time course was repeated twice (E2 and E3) at different microscope positions. Top panels = GCaMP-Ca^2+^ spiking activity, lower plots = non-spiking fluorescence.


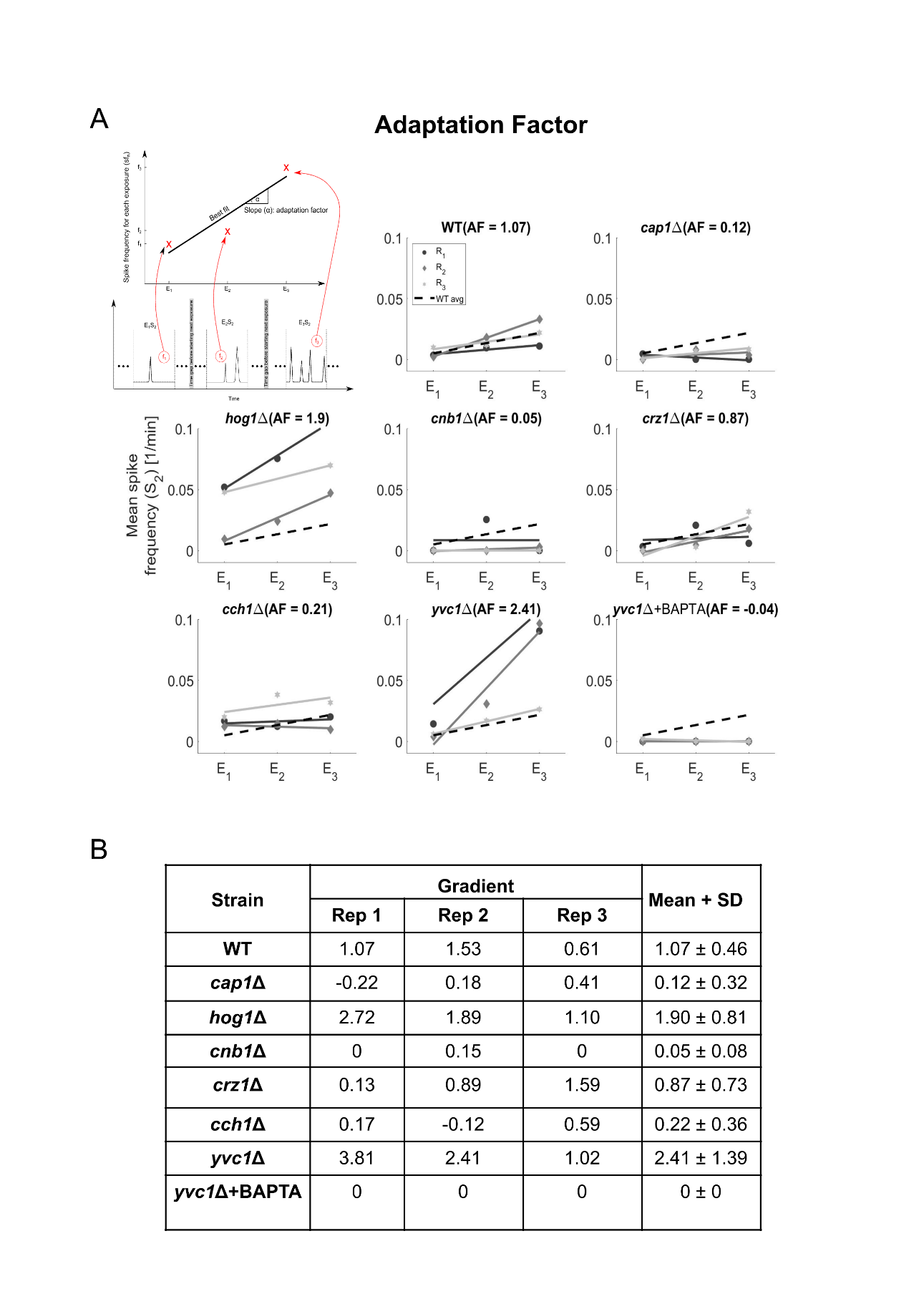


**Fig. S6: Adaptation Factors for wild-type and mutant strains exposed 3 times to 5 mM H_2_O_2_**

A. The ‘Adaptation Factor’ was defined as slope of the change in mean spike-rate from Exposure 1 to Exposure 3 (see illustration, top left panel). Plots showing the spike rate during exposure to H_2_O_2_) of WT and mutant strains. The line of best fit was plotted for each exposure. Each line represents an individual biological replicate. Dotted line = mean WT value (n = 3). B. Gradient values used to determine the mean gradient, ie the Adaptation Factor for each strain.


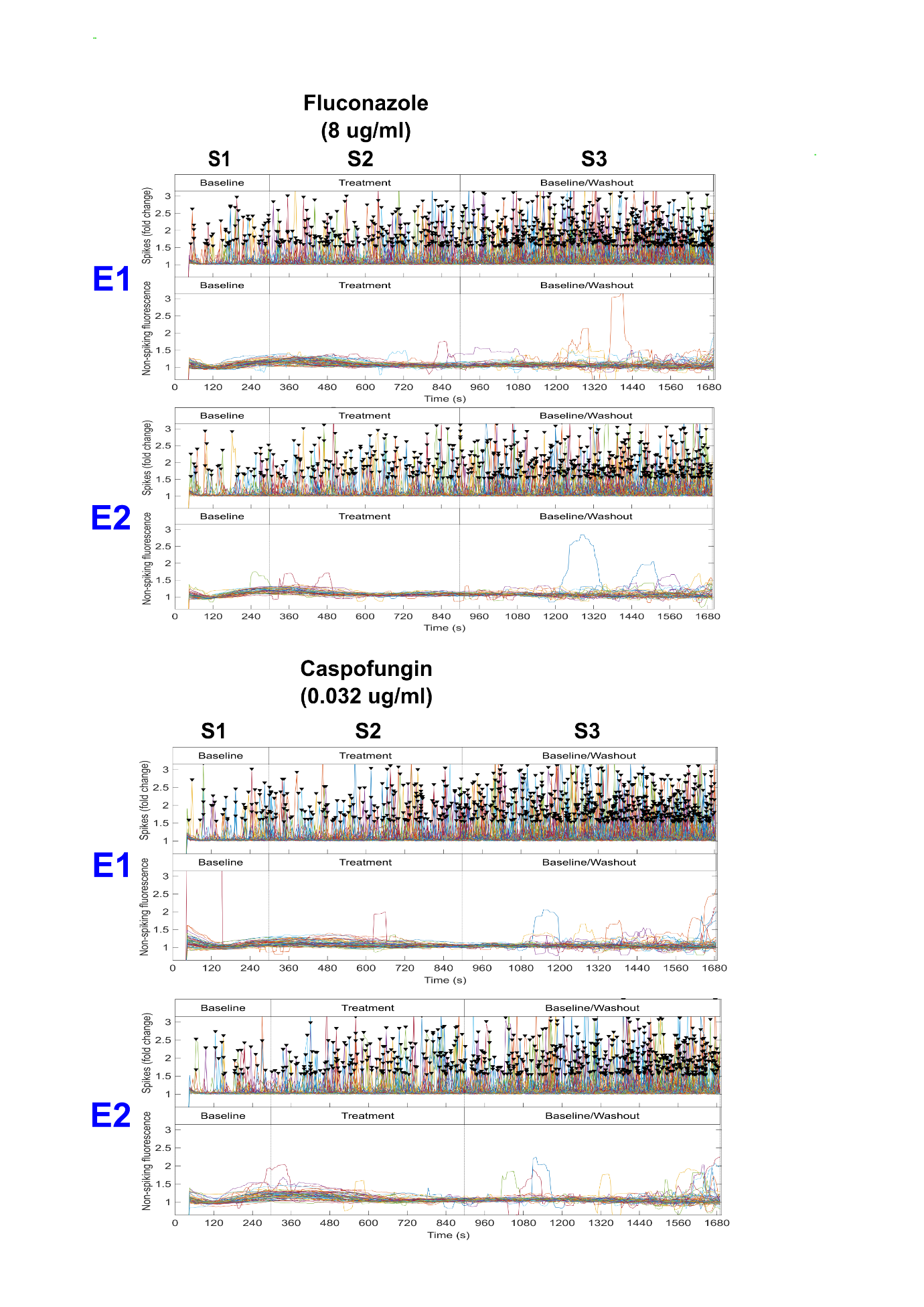


**Fig. S7: 10 min exposures to fluconazole or caspofungin do not affect Ca^2+^ GCaMP activity**. Spiking and non-spiking Ca^2+^ GCaMP plotss of wild-type cells exposed twice to 8 ug/ml fluconazole (top panels) or 0.032 ug/ml caspofungin (lower panels) during Stage 2.

**Supplementary references**

1. Fonzi WA, Irwin MY. 1993. Isogenic strain construction and gene mapping in *Candida albicans*. Genetics 134:717-728.

2. Wilson RB, Davis D, Mitchell AP. 1999. Rapid hypothesis testing with *Candida albicans* through gene disruption with short homology regions. Journal of Bacteriology 181:1868-1874.

3. Enjalbert B, Smith DA, Cornell MJ, Alam I, Nicholls S, Brown AJP, Quinn J. 2006. Role of the Hog1 stress-activated protein kinase in the global transcriptional response to stress in the fungal pathogen *Candida albicans*. Molecular Biology of the Cell 17:1018-1032.

4. Dantas ADS, Day A, Ikeh M, Kos I, Achan B, Quinn J. 2015. Oxidative stress responses in the human fungal pathogen, *Candida albicans*. Biomolecules 5:142-165.

5. Blankenship JR, Heitman J. 2005. Calcineurin is required for *Candida albicans* to survive calcium stress in serum. Infection and Immunity 73:5767-5774.

6. Karababa M, Valentino E, Pardini G, Coste AT, Bille J, Sanglard D. 2006. *CRZ1*, a target of the calcineurin pathway in *Candida albicans*. Molecular Microbiology 59:1429-1451.

7. Reedy JL, Filler SG, Heitman J. Elucidating the *Candida albicans* calcineurin signaling cascade controlling stress response and virulence. Fungal Genetics and Biology 47:107-116.

8. Barelle CJ, Manson CL, MacCallum DM, Odds FC, Gow NAR, Brown AJP. 2004. GFP as a quantitative reporter of gene regulation in *Candida albicans*. Yeast 21:333-340.

9. Shahana S, Childers DS, Ballou ER, Bohovych I, Odds FC, Gow NAR, Brown AJP. 2014. New Clox systems for rapid and efficient gene disruption in *Candida albicans*. PLOS ONE 9:e100390.

10. Chen TW, Wardill TJ, Sun Y, Pulver SR, Renninger SL, Baohan A, Schreiter ER, Kerr RA, Orger MB, Jayaraman V, Looger LL, Svoboda K, Kim DS. 2013. Ultrasensitive fluorescent proteins for imaging neuronal activity. Nature 499:295-300.
